# Mathematical models for assessing vaccination scenarios in several provinces in Indonesia

**DOI:** 10.1016/j.idm.2021.09.002

**Published:** 2021-09-24

**Authors:** N. Nuraini, K.K. Sukandar, P. Hadisoemarto, H. Susanto, A.I. Hasan, N. Sumarti

**Affiliations:** a Department of Mathematics, Institut Teknologi Bandung, Bandung, 40116, Indonesia; b Department of Public Health, Faculty of Medicine, Padjadjaran University, Jatinangor, 45363, Indonesia; c Department of Mathematics, Khalifa University, P.O. Box 127788, Abu Dabhi, UAE; d Department of Mathematical Sciences, University of Essex, Wivenhoe Park, Colchester, CO4 3SQ, United Kingdom; e Department of ICET and Natural Sciences, Faculty of Information Technology and Electrical Engineering, Ankeret, Ålesund, Larsgådsvegen 2, 6009, Norway

**Keywords:** COVID-19, *SIQRD* model, Age groups, Healthcare capacity, Vaccination strategy

## Abstract

To mitigate casualties from the COVID-19 outbreak, this study aims at assessing the optimal vaccination scenarios, considering several existing healthcare conditions and assumptions, by developing *SIQRD* (*Susceptible-Infected-Quarantine-Recovery-Death*) models for Jakarta, West Java, and Banten, in Indonesia. The models include an age-structured dynamic transmission model that naturally allows for different treatments among different age groups of the population. The simulation results show that the timing and period of the vaccination should be well planned and prioritizing particular age groups will give a significant impact on the total number of casualties.

## 1 Introduction

In March 2020, the World Health Organization declared the COVID-19 outbreak as a global pandemic. As of August 2021, it was reported that at least 206 million people have been infected worldwide, with death toll more than four million (COVID-19 coronavirus pandemic, 2020). From (Herawati and Setiyabudi, 2021) based on initial data of Indonesia, the infected people are mostly men (56.5*%*) and in the productive age, 31–59 years old (57.5*%*). Most deaths occurred at aged greater than 60 years (43.6*%*). The most recurrent clinical symptom was cough (77.8*%*), the most recurrent co-morbidity was hypertension (52.4*%*), and the province with the highest COVID-19 incidence was Jakarta (34.3*%*). The study in (Zakianis et al., 2021) reported that the average COVID-19 incident rate in Jakarta is 99.8 per 10,000 population. Risk factors for the spread of COVID-19 were associated with the population's high level of education, which can reflect a higher economic status to the population and a tendency to be more mobile.

By implementing well-planned and systematic mitigation strategies, including border closing and a mass vaccination, several countries have succeeded in suppressing the number of active cases. On the contrary, in Indonesia the number keeps increasing, even though the vaccination program has been implemented since early 2021. The death toll in Indonesia is around 115 thousands people (Kawal informasi seputar, 2020), which is the second largest number among Asian countries, after India (Indonesia’s coronavirus, 2020). Recently, the situation is even more frustrating due to the media news reporting some reinfection of COVID-19 (covid-19-coronavirus-vaccine, 2020), even though it has no clear evidence (Tang et al.,). Many presumed that it is due to the occurrence of new variants of the virus, that have been reported in some countries, including Brazil (Da Silva & Pena, 2021) and UK (Pan et al., 2021).

Inevitably, one way to mitigate the COVID-19 outbreaks is to develop effective vaccines and produce them massively for all affected countries. Vaccine could prevent a susceptible person from being infected at least for a time period or even for a lifetime. In early 2021, Indonesia has started a program of first jab with Sinovac, a COVID-19 vaccine developed by a China-based biopharmaceutical company, which has been tested in Indonesia for a Phase 3 clinical trial (Sinovac launches Phase 3, 2020). This program has brought a fresh hope for COVID-19 mitigation in Indonesia, even though resistance still occurs in some areas.

However, the quick need for vaccination is like a double-edged sword. Due to the history of other virus-induced illnesses, as well as issues of vaccine mismatch and suspected side effects on immunocompromised individuals, vaccine administration should be well observed. Instead of suppressing the morbidity and mortality of COVID-19, a lack of consideration on vaccination scenarios could also cause unwanted results, such as COVID-19 s outbreak, ineffective vaccination, etc. These pose challenges to the policy-makers and researchers to consider the best scenario of vaccination.

Recently, several studies have been conducted that model COVID-19 vaccination. (Bitsouni et al., 2020) discussed the influence of vaccine effectiveness using a *SEIAR* (*Susceptible-Exposed-Infected-Asymptomatic-Recovered*) model based on cases in Italy. The study focused on the risk of infection spread, the peak prevalence of infection and the time at which the peak prevalence occurred. The paper by Zindoga et al. (Mukandavire et al., 2020) estimated the effect of social distancing implementation and explored vaccine efficacy scenarios based on cases in South Africa. (Lin et al., 2020), proposed a mathematical model to understand the spread of the virus as the response to the individual behavioral reaction and governmental action, such as travel restriction and quarantine.

In order to describe the behavior of COVID-19 spread in several provinces of Indonesia, i.e. Jakarta, Banten, and West Java, we propose mathematical models, based on non-age-structured and age-structured SIQRD (*Susceptible-Infected-Quarantine-Recovery-Death*) models. The level of effectiveness of the vaccination program is put into the model by considering the proportion of those who likely to recover or be immune to the illness after getting vaccinated. Simultaneously, there is a chance of reinfection by allowing a portion of the recovered people to be re-infected after a particular time. We assume that this re-infection can be caused by the same or different variant of viruses. By using these constructed models and simulation scenarios, we propose an estimation of optimum vaccination schedule considering the vaccination cost, healthcare capacity, and vaccination capacity per day. Based on the simulation, the effect of the timing to begin the vaccination on the mortality and the morbidity cases, is also observed. Eventually, we consider several scenarios in prioritizing different age groups and evaluate the results.

The major questions we would like to address in this paper are:

(Q1) Which mathematical model best represents the COVID-19 spread in Jakarta, Banten and West Java of Indonesia? Does the reinfection of recovered people cause the increase of spreading significantly?
(Q2) What is the effect of vaccination on mortality and morbidity cases of all age groups? When is the optimal timing to begin the vaccination?
(Q3) Can we find an optimal vaccination scenario considering the existing government's capacities, including the healthcare facility, and estimated maximum capacity of vaccination per day? How many people should be vaccinated for mitigating the spread?
(Q4) If we consider the age-structure of susceptible people in those provinces, should we prioritize some age groups over the others?

The answers to these questions may help the policymaker, especially in Indonesia, for planning a vaccination program in the near future.

## 2 Proposed model and analysis

In this section, we modify the standard *SIRD* model, that has been commonly used in modeling vaccination of other virus-induced illnesses, such as influenza (Feng et al., 2011; Hethcote & Waltman, 1973; Qian et al., 2020; Tasmi, 2016). The obtained models, *SIQRD*, representing the impacted populations due to COVID-19 spread are constructed with and without age structures. The modification includes the addition of the compartment *Q* representing the dynamics of quarantined people by reckoning the importance of governmental action (Lin et al., 2020). The relation among variables are necessarily constructed based on the observation of the real examined problem.

### 2.1 Non-age-structured model

**Fig. 1:**
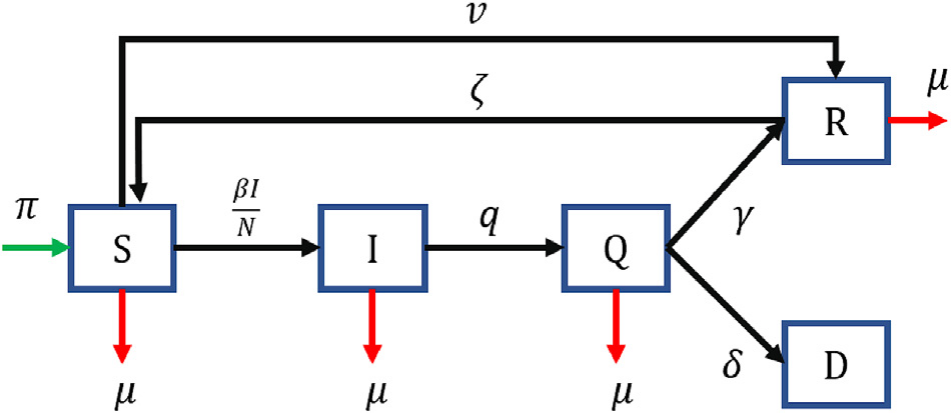
Flow diagram of the SIQRD model. Blue and red arrows represent respectively the natural recruitment and death rate.

Using the flow diagram in Fig. 1, the vaccination program transfers the suspected people directly into compartment *R*. The vaccination scenarios will be accommodated by determining a particular function of *v*(*t*). Some people in *R*, the recovered and vaccinated people, possibly can be infected again, so they are transferred back to *S* with transfer rate *ζ*. Although there is no scientifically clear evidence of reinfected cases of COVID-19 according to (Tang et al.,), yet it is important to follow-up the recovered population for being reinfected to prevent a further spread. There is a possibility that reinfection could be caused by new variants of viruses. On the other hand, the vaccine may not give the perfect protection from infection due to a mismatch problem of the type of virus being used for the vaccine. We can assume that the susceptible people are infected only due to their contact with people in compartment *I*. The hospitalization or successful isolation of the quarantined ones in compartment *Q* will not cause infection. We define compartment *R* as the total number of immune people due to both full recovery from the illness and taking vaccination.

Using the description of variables and parameters in Table 1, the constructed mathematical model is given as follows.

(1) $$\begin{array}{ccc} \frac{dS\left(t\right)}{dt} & = & \pi \hat{N}\left(t\right)+\zeta R\left(t\right)-\left(\beta \frac{I\left(t\right)}{N}+\eta v\left(t\right)+\mu \right)S\left(t\right),\\ \frac{dI\left(t\right)}{dt} & = & \frac{\beta S\left(t\right)I\left(t\right)}{N}-\left(q+\mu \right)I\left(t\right),\\ \frac{dQ\left(t\right)}{dt} & = & qI\left(t\right)-\left(\gamma +\delta +\mu \right)Q\left(t\right),\\ \frac{dR\left(t\right)}{dt} & = & \gamma Q\left(t\right)+\eta \upsilon \left(t\right)S\left(t\right)-\left(\zeta +\mu \right)R\left(t\right),\\ \frac{dD\left(t\right)}{dt} & = & \delta Q\left(t\right). \end{array}$$

in which system (1) satisfies *N* = *S*(*t*) + *I*(*t*) + *Q*(*t*) + *R*(*t*) + *D*(*t*). Defining $\hat{N}\left(t\right)=N-D\left(t\right)$ that represents the number of living people that will generate susceptible newborns, we have

$$\frac{dN}{dt}=\left(\pi -\mu \right)\hat{N}\left(t\right).$$

**Table 1:** Description of variables and parameters.

Notations	Description	Unit	Value
*S*(*t*)	Number of susceptible population at time-*t*	person	–
*I*(*t*)	Number of infectious population yet not quarantined at time-*t*	person	–
*Q*(*t*)	Number of infectious population but being quarantined at time-*t*	person	–
*R*(*t*)	Number of immune population at time-*t*	person	–
*D*(*t*)	Number of deceased population at time-*t*	person	–
*β*	Transmission rate	1/day	estimated
*γ*	Recovery rate due to COVID-19	1/day	estimated
*δ*	Death rate due to COVID-19	1/day	estimated
*q*	Quarantine rate	1/day	assumed
*v*(*t*)	Vaccination rate	1/day	calculated
*η*	Vaccine efficacy	–	assumed
*ζ*	Reinfection rate	1/day	assumed
*μ*	Natural recruitment (birth) rate	1/day	assumed
*π*	Natural death rate	1/day	assumed

For the sake of simplicity, the number of population is assumed to be constant over the time horizon, or $\frac{dN}{dt}=0$, so we have *π* = *μ*.

In this model, there are viral-related and intervention-related parameters and their values are assumed to be constant. The former ones consist of transmission rate, recovery and death rates due to COVID-19 and reinfection rate. Besides the natural birth and death rates, the remaining parameters are considered to be the intervention-related parameters. The values of some of the parameters are given by assumption that follow the existing findings. The natural birth and death rates are obtained from the statistical data of the provinces. The quarantine rate is based on the time needed for a person to be not-infectious anymore to other persons. The vaccine efficacy and reinfection rate are given, but the values can be simulated in order to see the dynamic of the variables' changes. Other parameter values are estimated/calculated as the results of the implemented numerical method, so that graphs of variables being observed will be the closest that the method can get to the real data provided for each province. Further explanation of these parameters’ estimation is given in Section 4.1.

In the constructed models, the vaccination process impacts on a direct transfer of people from compartment *S* to compartment *R* due to the emerging of immune system inside the body of the vaccinated people. To develop the vaccination process, we design a periodical schedule, where the vaccination was given several times at certain timings and its value rate will be constant between two timings. There will be *k* times of vaccination program so there will be *k* periods from the beginning of the scenario. Here simply we assume that the jab needed is once per individual, whereas two-jab vaccination can be accommodated in the model further by only adjusting its parameter values.

Having determined the above definition, *υ*(*t*) will be a piecewise function that has constant values during each period of time. The mathematical formulation of vaccination rate *v*(*t*) is as follows:

(2) $$v\left(t\right)=\left\{\begin{array}{ll} 0,\; & t_{0}\leq t<t_{v}\\ c_{1},\; & t_{v}\leq t<t_{v}+\Delta \\ \ldots \;\\ c_{k},\; & t_{0}+\left(k-1\right)\Delta \leq t<t_{V} \end{array}\right)$$

where *c*_*j*_ is a positive constant, *t*_0_ is the initial time of the pandemic, *t*_*v*_ is the time of the first vaccination shot, *t*_*V*_ = *t*_*υ*_ + *k*Δ is the end of *k*-th vaccination period, and Δ (in days) is the length of each period that has the same rate of inoculation. Firstly, the constructed model is simulated within the first interval when there is no vaccination, or *v*(*t*) = 0, and the result will become the reference to make the comparison to the other periods of the vaccination program. In this paper, the rate of vaccination *c*_*i*_, *i* ∈ {1, 2, …, *k*}, during the *i*-th time interval, is calculated by solving an optimization problem based on some healthcare aspects existing in Indonesia. The detailed description of the optimization problem is explained further in Section 5.1.

### 2.2 Age-structured model

The age-structured model of *SIQRD* is similar to the previous system of Eq. (1). For each compartment, we define five age groups, i.e. 0-9, 10–19, 20–49, 50–59, and 60 or higher, so all age groups have a compartment assigned to them. For age group *i* ∈ {1, 2, 3, 4, 5}, the obtained model is given as.

(3) $$\begin{array}{ccc} \frac{dS_{i}\left(t\right)}{dt} & = & \pi \hat{N_{i}}\left(t\right)+\zeta R_{i}\left(t\right)-\left(\underset{j}{\sum }\left(\beta_{ij}\frac{I_{j}\left(t\right)}{N}\right)+\eta v_{i}\left(t\right)+\mu \right)S_{i}\left(t\right),\\ \frac{dI_{i}\left(t\right)}{dt} & = & \underset{j}{\sum }\left(\beta_{ij}\frac{I_{j}\left(t\right)}{N}\right)-\left(q+\mu \right)I_{i}\left(t\right),\\ \frac{dQ_{i}\left(t\right)}{dt} & = & qI_{i}\left(t\right)-\left(\gamma +\delta +\mu \right)Q_{i}\left(t\right),\\ \frac{dR_{i}\left(t\right)}{dt} & = & \gamma Q_{i}\left(t\right)+\eta v_{i}\left(t\right)S_{i}\left(t\right)-\left(\zeta +\mu \right)R_{i}\left(t\right),\\ \frac{dD_{i}\left(t\right)}{dt} & = & \delta Q_{i}\left(t\right). \end{array}$$

where $\hat{N_{i}}\left(t\right)=N_{i}-D_{i}\left(t\right)$, and *N*_*i*_ = *S*_*i*_(*t*) + *I*_*i*_(*t*) + *Q*_*i*_(*t*) + *R*_*i*_(*t*) + *D*_*i*_(*t*). Vaccination rate *v*(*t*) given by Eq. (2) is also implemented into this age-structured model where the vaccination rate *v*_*i*_(*t*) refers to the vaccination rate of the age group *i*. For example, *υ*_2_(*t*) is defined the vaccination rate of the 2^*nd*^ age group, where values *c*_*ij*_ are positive constants, for *i* = 1, 2, *…*, 5 and *j* = 1, 2, *…*, *k*.

In the first and second equations of system (3), the transmission rate *β*_*ij*_ = *β*_*i*_*C*_*ij*_ gives cross transmission between age groups *i* and *j*, where *β*_*i*_ represents the probability of infection level in age group *i*, and *C*_*ij*_ is a matrix describing contacts between any pair of age group *i* and *j*, where *i*, *j* = 1, 2, *…*, 5. Here the unit of *C*_*ij*_ is 1/day. Function *v*_*i*_(*t*) gives the potent vaccination rate of group *i*, which shows the success of vaccination program based on its efficacy for the age group *i*. We define the values of *C*_*ij*_ based on the results from the study in (Mossong et al., 2008), while the values of *β*_*i*_ are estimated based on the real data provided in Indonesia.

## 3 Datasets

The data of COVID-19 in Jakarta, Banten, and West Java are retrieved from https://kawalcovid19.id/(Kawal informasi seputar, 2020). It consists of time-series data of active cases, total recovered cases, and total deaths from late March until late October 2020, which we call as *Dataset 1* following which will be used to extract the parameters of the systems (1) and (3). The chosen time period of the data is expected to represent the real figure of COVID-19 spread in each province before the vaccine inoculation. On the other hand, it is unfortunate that the data of COVID-19 victims along the observed time period do not include details of their age. We define values of the parameter *β*_*i*_ for system of Eq. (3) from comparing the population pyramid of a state in the USA that has the desired data and similar portions of age groups with that of the provinces being observed. The best choice is Connecticut, USA, where its data resembles enough the provinces data. The COVID-19 data of Connecticut, USA, is retrieved from https://data.ct.gov/(COVID-19 cases and deaths by age group, 2020). This website provides time-series data on total infections by age group, which we will call as Dataset 2. The ratio between the number of COVID-19 victims and the total number of population per age group in each province is assumed to be the same with that in Connecticut. Data on population by age group in Jakarta, Banten, West Java, and Connecticut are retrieved from (Jumlah Penduduk Menurut, 2020; Pusat Data Ekonomi, 2020) and (Connecticut's official state website, 2020).

## 4 Numerical results

### 4.1 Parameters estimation

Firstly, we introduce two additional variables; *CR*(*t*), representing the total number of recovery due to COVID-19, and *V*(*t*), depicting the total figure of vaccine inoculation at time_*t*. The rate of the first variable is proportional to the existing number of people in *Q*, or

$$\frac{dCR\left(t\right)}{dt}=\gamma Q\left(t\right).$$

In this stage, the transmission, recovery, and death rates due to COVID-19 are estimated numerically using the Least Square Method (LSM), so that the values of *Q*(*t*), *CR*(*t*) and *D*(*t*) generated from system (1) are close enough to the respective real data of active cases (*DATA*_*Q*_), total recovered (*DATA*_*CR*_), and total death (*DATA*_*D*_) due to COVID-19. The initial value of compartment *I*(*t*) is unknown since it also includes the non-quarantined ones. We therefore estimate its value. Simultaneously, we define

$$T=\left[\begin{array}{c} \beta \\ \gamma \\ \delta \\ I\left(t_{0}\right) \end{array}\right],$$

where their values are the solution of the following minimization problem.

(4) $$\underset{T\in D}{\min}\overset{N}{\underset{j=1}{\sum }}\left(\Delta_{Q}{\left(t_{j},T\right)}^{2}+\Delta_{CR}{\left(t_{j},T\right)}^{2}+\Delta_{D}{\left(t_{j},T\right)}^{2}\right)$$

with $\Delta_{Q}\left(t_{j},T\right)=Q\left(t_{j},T\right)-DATA_{Q}\left(t_{j}\right),\Delta_{CR}\left(t_{j},T\right)=CR\left(t_{j},T\right)-DATA_{CR}\left(t_{j}\right),\Delta_{D}\left(t_{j},T\right)=D\left(t_{j},T\right)-DATA_{D}\left(t_{j}\right)$ and D being the search domain. Remember that *DATA*_*Q*_, *DATA*_*QR*_, *DATA*_*D*_ are the actual retrieved data. Basically, the best estimated value of T makes the objective function given by Eq. (4) close to zero, which means that the model dynamics is close to the real data.

The second variable is defined following this differential equation

(5) $$\frac{dV\left(t\right)}{dt}=\upsilon \left(t\right)S\left(t\right).$$

Note that the number of vaccinated people *V*(*t*) at time *t* is not always fully added up to the number of immune people *R*(*t*) because of the effect of vaccine efficacy. The values *V*(*t*) are computed as the results of solving Eq. (5) together with the SIQRD model, using the built-in function ode45 in MATLAB.

In the fitting process, the natural recruitment and the natural death rates are $\pi =\mu =\frac{1}{365\cdot 70}$ where the life expectancy in Indonesia is 70 years, based on (Indonesia: Life Expectancy, 2020). The other assumed values of parameters are *q* = 0.4 (the quarantine rate), $\zeta =\frac{1}{150}$ (reinfection rate) according to (Gudbjartsson, 2020), and *v*(*t*) = 0 for *t* in a period when the vaccine is not available. Later, we set *η* = 80*%* (the vaccine efficacy) when the vaccine is available.

**Fig. 2:**
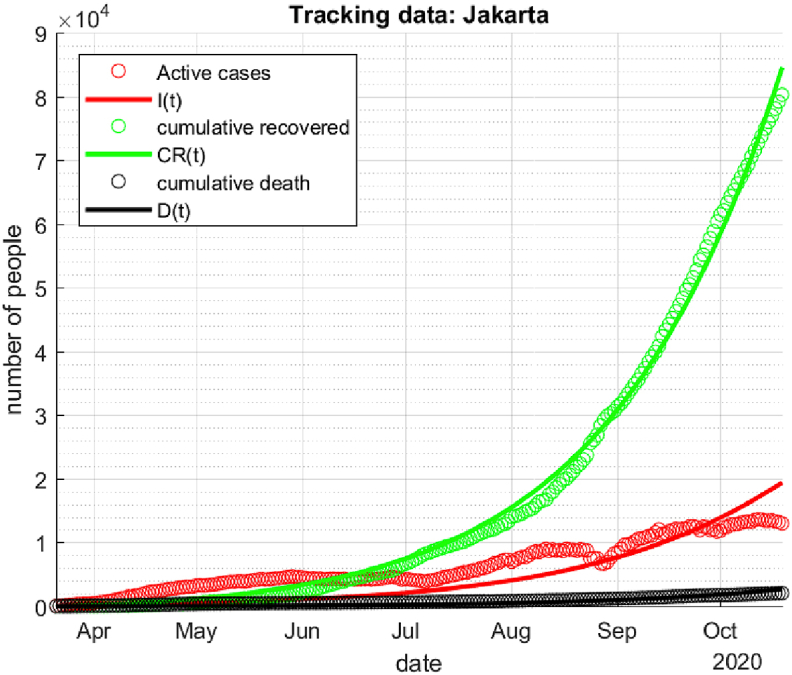
Fitting result of the non-age-structured model for Jakarta.

As in Table 2 for systems (1) and (3), the estimated values of parameters and initial condition of *I*(*t*) in Jakarta are given. Based on (Kapan Sebenarnya Corona, 2020), there were only 2 cases reported in the first day of pandemic in Jakarta. Using the Jakarta's basic reproduction number that is slightly higher than 1, approximately there were more than 60 persons infected that day. The simulation of the model utilizing the estimated parameters and initial condition is depicted in Fig. 2 plotted together with the existing data. The dynamics of total recovered and deceased due to COVID-19 are well-fitted to the data, although the figure for total deceased is extremely smaller than that of recovered. However, particularly in Jakarta, the active case dynamic is close-fitted to the data only in the late simulation since it underestimates the fluctuating data at other times. Given the assumption of constant parameters over time, the model is not able to capture the data precisely, especially when they fluctuate. However, the data are well-fitted in most of the observed time in Jakarta. The other two fitting results for Banten and West Java are given in Appendix A, where the model also fit the data most of the time.

**Table 2:** Parameter estimation of the non-age-structured model for Jakarta.

*β*	*γ*	*δ*	*I*(*t*_0_)	*R*_0_
0.4215	0.0876	0.0028	66	1.0134

To construct the age structured model in system of Eq. (3) for each province, we apply the Connecticut data by using Algorithm 4.1. Note that we use the same values of *β*, *γ*, *δ* and *I*(*t*_0_) from the non-age-structured model for each province.

Algorithm 1 Estimating the values of *β*_*i*_

**Figure:**
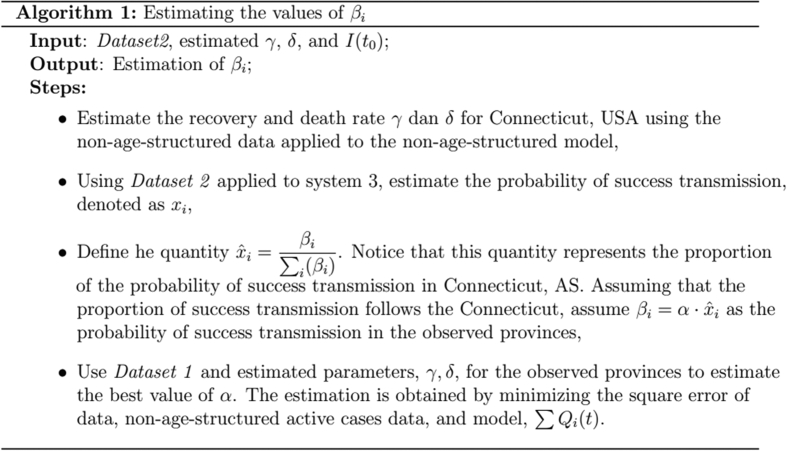

**Fig. 3:**
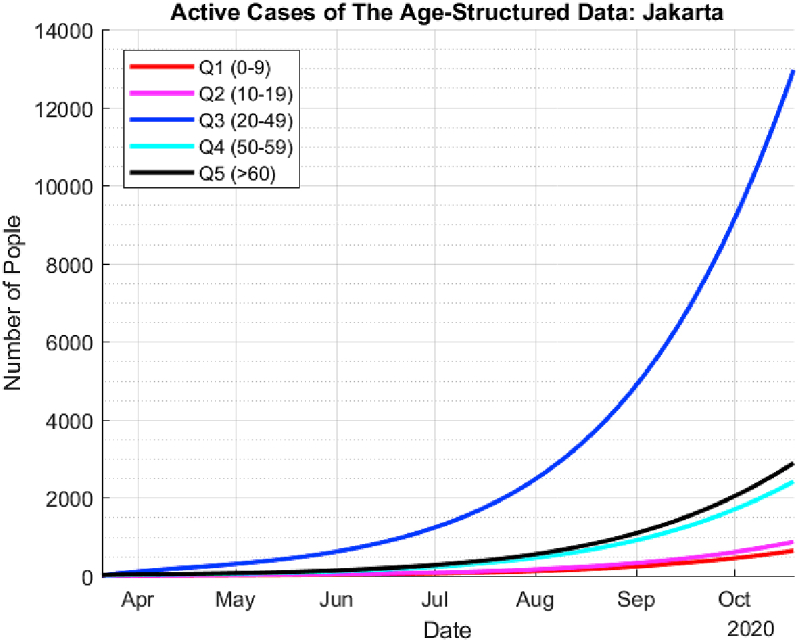
Estimated Active Cases among age groups for Jakarta.

The obtained estimation of *β*_*i*_ of Jakarta, for *i* ∈ {1, 2, 3, 4, 5} are shown in Table 3. The largest values are owned by age-groups 3,4, and 5 using Algorithm 4.1. The numbers of active cases in Jakarta are dominated by three older age-groups as seen in Fig. 3. The results for other provinces are available in the Appendix.

**Table 3:** Obtained values *β*_*i*_ for the age-structured model of Jakarta.

*β*_1_	*β*_2_	*β*_3_	*β*_4_	*β*_5_
0.0251	0.0355	0.1122	0.1698	0.4488

### 4.2 Dynamics of models with and without vaccination

**Fig. 4:**
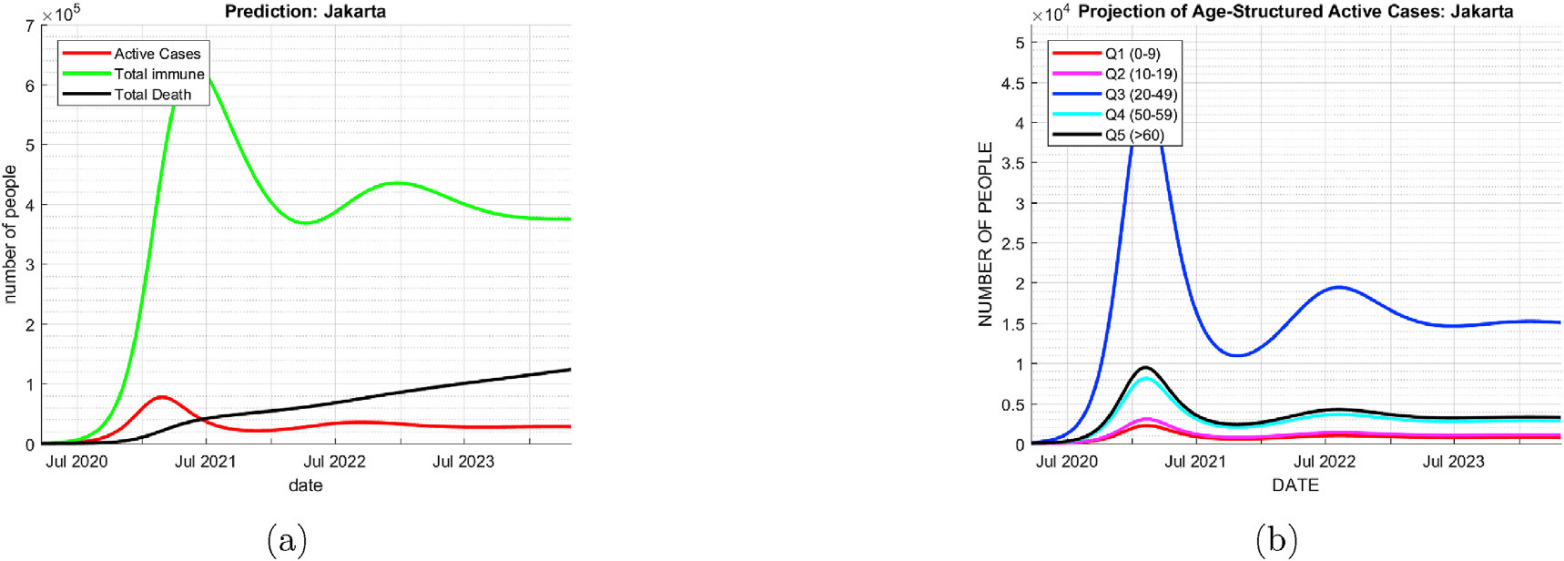
SIQRD simulations without vaccination in Jakarta: (a) non-age-structured; (b) age-structured model.

Having developed the *SIQRD* model with and without age classes, first we analyze the dynamics of the models for *υ*(*t*) = 0 or without vaccination to project the peak of the outbreak that may happen in the near future. Fig. 4(a) portrays the dynamics of *Q*(*t*), *R*(*t*) and *D*(*t*) for the non age-structured model of Jakarta. It shows that the peak of active cases will be in February 2021. On the other hand, the number of immune people will largely increase and then decrease as some of them become susceptible again. This condition is possibly causing the second outbreak of the disease later. Notice that for a certain region, the result given by the non-age-structured model is similar to at given by the age-structured one, e.g. in the peak occurrence time. However, they are not precisely the same in numbers since we use different objective functions on estimating its parameters. For instance, both Fig. 4(a) and (b) are showing that the peak of outbreak will occur in February 2021 in Jakarta. However, they are numerically different since the non-age-structured model estimated that the number of active cases reaches nearly 80,000 cases while the age-structured one estimated 65,000.

In Fig. 4(a), the graph of the total number of deaths *D*(*t*) increases because of the existence of infectious people. Based on the age-structured model, the dynamics of active cases in Jakarta are classified into 5 age groups in Fig. 4(b). Similar to Fig. 4(a), the age-structured model also illustrates the second outbreak due to the reinfection for each age-groups. By using the constructed model, this simulation shows that reinfection played a vital role in producing the second outbreak.

**Fig. 5:**
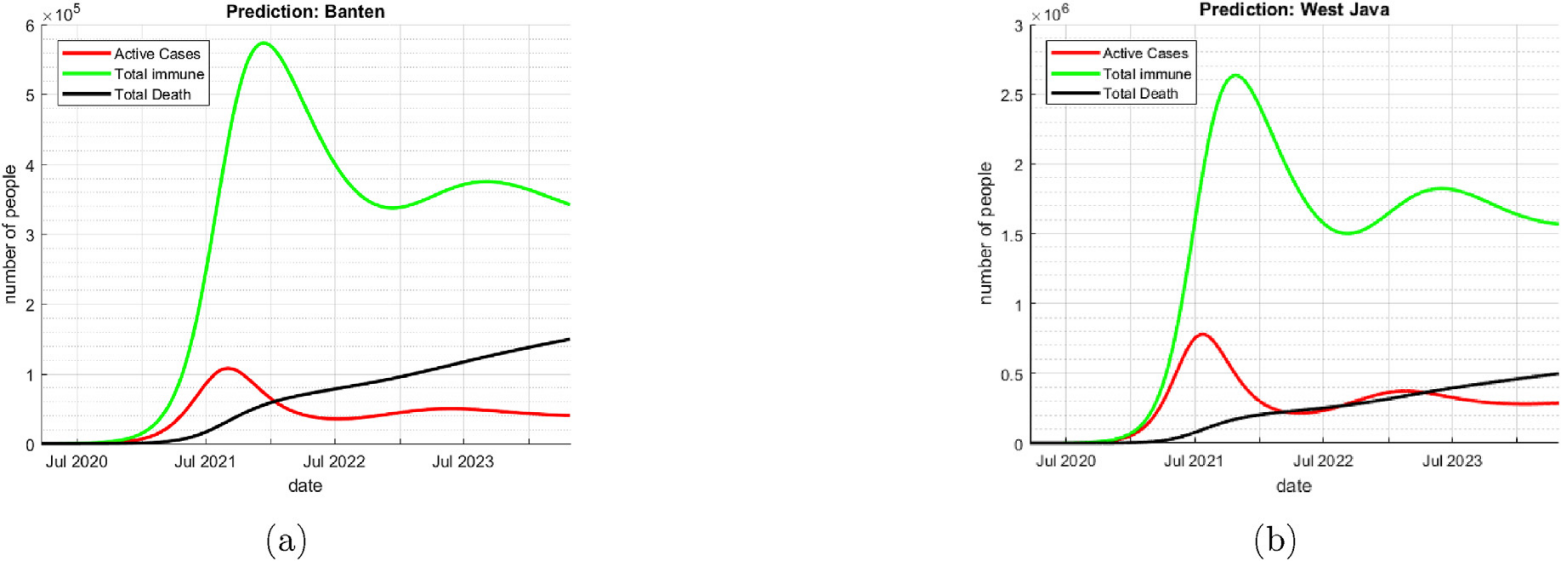
SIQRD simulations without vaccination in: (a) Banten; (b) West Java.

The peak of outbreak projections of SIQRD model in Banten and West Java are given in Fig. 5. Different from the projection of Jakarta, the dynamics of active cases will increase until August 2021. This is possible because the populations of Banten and West Java are larger than that of Jakarta. Nevertheless, the general behavior of the model is similar where the reinfection factor could cause the second peak of outbreak.

**Fig. 6:**
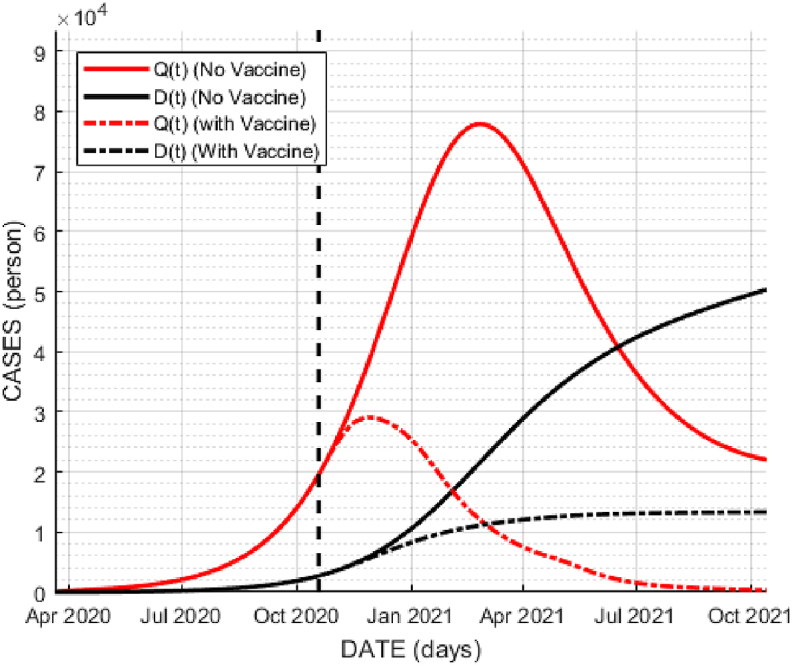
Dynamics of the SIQRD model with vaccination applied in Jakarta.

Now we discuss whether or not the presence of vaccination will generally reduce the number of active cases and total deaths. Based on Eq. (2), we set *k* = 12 and the length of one period was 30 days, so the whole vaccination period is about one year. Set *c*_*j*_ to be any values randomly chosen, with 0 ≤ *c*_*j*_ ≤ 10^−3^, which represents the vaccination rate in the *j*th-period, *j* = 1, 2, *…*, *k*. The vaccination program is set to begin in the third week of October 2020. It is shown in Fig. 6, that the numbers of active cases and total deaths after 1–2 months of vaccination in Jakarta become less than half of the numbers in the model without vaccination.

Later in Section 5, we will determine the optimal values of *v*_*i*_(*t*) that met some desired constraints. There will be several scenarios of vaccination being assessed in particular age groups so we could analyze the urgency of a vaccination program.

### 4.3 Sensitivity analysis on vaccine efficacy and quarantine rate

**Fig. 7:**
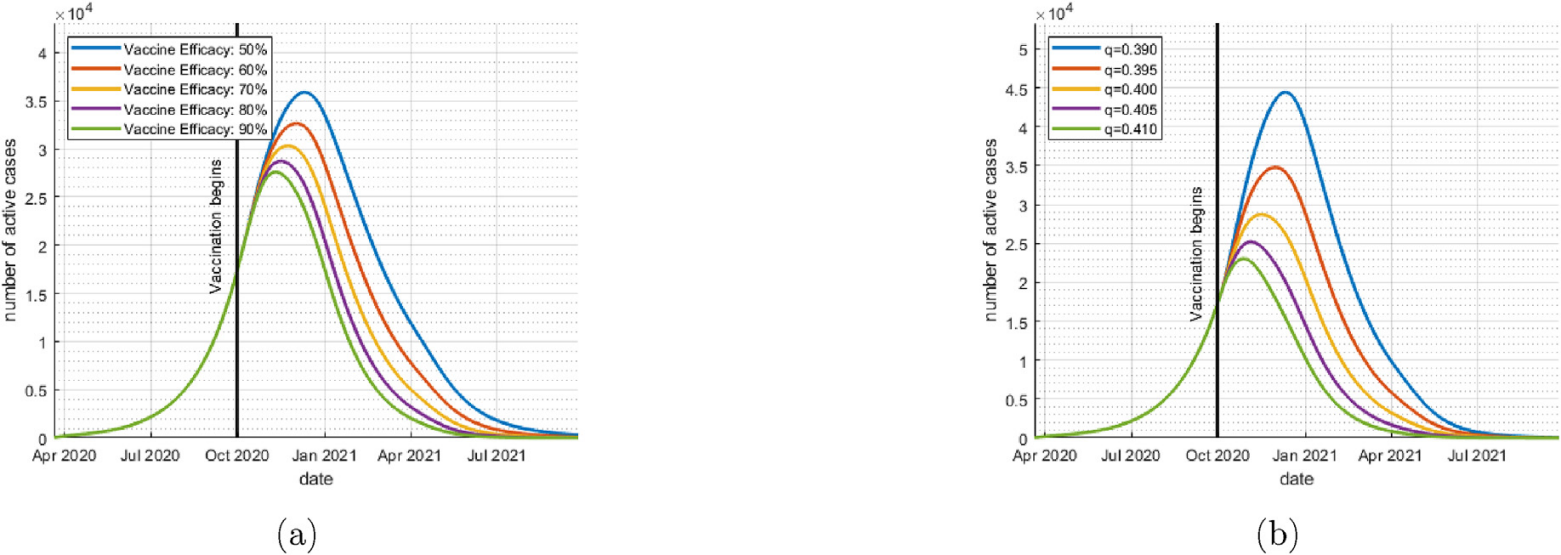
Dynamics of active cases with several values of vaccine efficacy and quarantine rate.

We want to observe the dynamics of the active cases *Q*(*t*) using simulations with different values of vaccine efficacy *η* and quarantine rate *q*. The first simulation is done by varying the vaccine efficacy and keeping the quarantine rate constant, where the results are in Fig. 7(a). The second simulation is using the opposite scheme, where the results are in Fig. 7(b). The values for *υ*(*t*) are chosen to be the same as the ones already used in the previous subsection.

Fig. 7(a) depicts the change in the numbers of active cases as the vaccine efficacy varies. The greater the vaccine efficacy, the more effective the vaccination is in suppressing the number of active cases. Remember that $\hat{v}\left(t\right)=\eta v\left(t\right)$. When the vaccine efficacy is low, we require high values of *v*(*t*) to suppress the numbers of mortality and morbidity of COVID-19. Fig. 7(b) shows that the higher the value of the quarantine rate, the lower the number of active cases is. As a conclusion, we need a vaccine with as high efficacy as possible, and a quite high quarantine rate to suppress the number of active cases. The total number of deaths will follow the same pattern.

## 5 Several scenarios of vaccination

Previously, the simulations were carried out without any constraints in finding the solutions of SIQRD systems (1) and (3). Now we examine several scenarios for vaccination with the main objective of effectively reducing the numbers of active cases and death toll at the minimum cost. It is obvious that high vaccination rate needs a large number of vaccines provided by the government, and as such it requires high expenses. We establish an approximation problem dealing with the vaccination function *v*(*t*) with some constraints based on the real situation. There are conditions that should be considered in this optimization process in order to have a feasible solution for the real problem, which are as follows:

• The maximum number of active cases does not exceed the maximum capacity of the existing healthcare facility, denoted by *K*_1_ (persons),
• The number of daily vaccine does not exceed the maximum shots of vaccination per day provided by the Indonesian government, denoted by *K*_2_ (per day).

The model being observed first is the non age-structured one. Some of the results of this optimization problem using this model will be used to execute scenarios using the age-structured model later. Since comorbidities contribute to a high number of deaths, we assume that they exist in all age groups. Thus, applying vaccines to all age groups simultaneously can be one of the scenarios worth considering. The scenarios are implemented to find the answer to questions (Q1)-(Q3).

### 5.1 Non age-structured model

In accordance with the required constraints, we define the optimization problem as follows:

Minimize

(6) $$f=\omega \overset{k}{\underset{m=1}{\sum }}\int_{t_{v}+\left(m-1\right)\Delta }^{\left(t_{v}+m\Delta \right)}v\left(t\right)S\left(t\right)dt$$

where *t* ∈ [*t*_*v*_, *t*_*V*_] is defined in Section 2.1 and *ω* represents the cost needed to vaccine a single person. Besides that, the objective function *f* has to meet the following constraints:

• max_*t*_*Q*(*t*) ≤ *K*_1_,
• For *t* ∈ {*t*_*v*_, *t*_*v*_ + 1, *t*_*v*_ + 2, …, *t*_*V*_ − 1}, $\int_{t}^{t+1}v\left(t\right)S\left(t\right)dt\leq K_{2}$.
• *v*(*t*) ≥ 0 for *t* ∈ {*t*_*v*_, *t*_*v*_ + 1, *t*_*v*_ + 2, …, *t*_*V*_}

As Δ represents the time interval of each vaccination, it is defined as 30 days, which means that the rate of vaccination will be kept to level off in certain values for every 30 days in a row. This assumption is based on the initial plan of the government on providing the vaccine in the term of months. According to (Biofarma, 2021) considering the readiness of the vaccine, the first vaccine inoculations are set in several phases having different length periods, e.g. 4 months (Jan–Apr 2021) for the first phase and 3 months later (May–Jul 2021) for the second phase of vaccination. Hence, choosing Δ = 30 days is reasonable and it allows the rates of vaccine to vary even though they are in the same phase of vaccination. For the maximum number of active cases *K*_1_ and the maximum number of vaccinated persons *K*_2_, we define two possible conditions; limited and good facilities, and analysed whether there are solutions for this approximation or not. Practically, the rate of vaccination *v*(*t*) is obtained by means of solving the defined optimization problem. The original constrained problem is first converted into an unconstrained one by adding a penalty value once the point violates the constraints following which it is solved numerically using the trust-region method.

According to (Pemerintah RI Targetkan, 2020), the Indonesian government is able to provide about 31,000 COVID-19 testing devices per day nationwide. However these testings kits are not evenly distributed to all provinces in Indonesia. Respectively, Jakarta, Banten and West Java get approximately 31%, 3.4%, and 10% of the total number of provided testing kits. We assume the limited facility condition based on this news, where the maximum numbers of vaccines received by Jakarta, Banten and West Java are 10,000, 1,100, and 3,100 vaccines per day, respectively. On the other hand, the government claimed that there will be 1 million vaccines per day nationwide (Indonesia masih kejar, 2020). We assume the good facility condition based on this news, so the maximum availability of vaccines are 310,000 vaccines for Jakarta, 100,000 vaccines for West Java, and 34,000 vaccines for Banten.

The model using the assumption of limited and good conditions of facility has been examined with the vaccination starting in October 2020. Solving the optimum process problem with limited facility condition, we could not find any solution. Therefore, the good facility condition is used for simulations from now onward. In showing the results, the output graph *D*(*t*) and *Q*(*t*) are always compared with that coming from the model without vaccination so we can see their stark differences.

#### 5.1.1 Starting times of vaccination

We set the vaccination program to start at different times: October 2020, January 2021 and after the peak of outbreak for each province by setting different *t*_*v*_ for each simulation. The three scenarios are aimed to compare the expected situation if the first vaccine jab is delivered at different times. The value *K*_1_ is adjusted for each province to be higher than the existing number of active cases at the time when the vaccination starts. The values of *K*_1_ and *K*_2_ are given in Table 4.

**Table 4:** Values of *K*_1_ and *K*_2_ for each province.

Times of vaccination	Jakarta		Banten		West Java	
	*K*_1_	*K*_2_	*K*_1_	*K*_2_	*K*_1_	*K*_2_
October 2020	30,000	310,000	20,000	34,000	110,000	100,000
January 2021	65,000	310,000	20,000	34,000	110,000	100,000
After the peak of outbreak	78,500	310,000	100.000	34,000	780,000	100,000

**Fig. 8:**
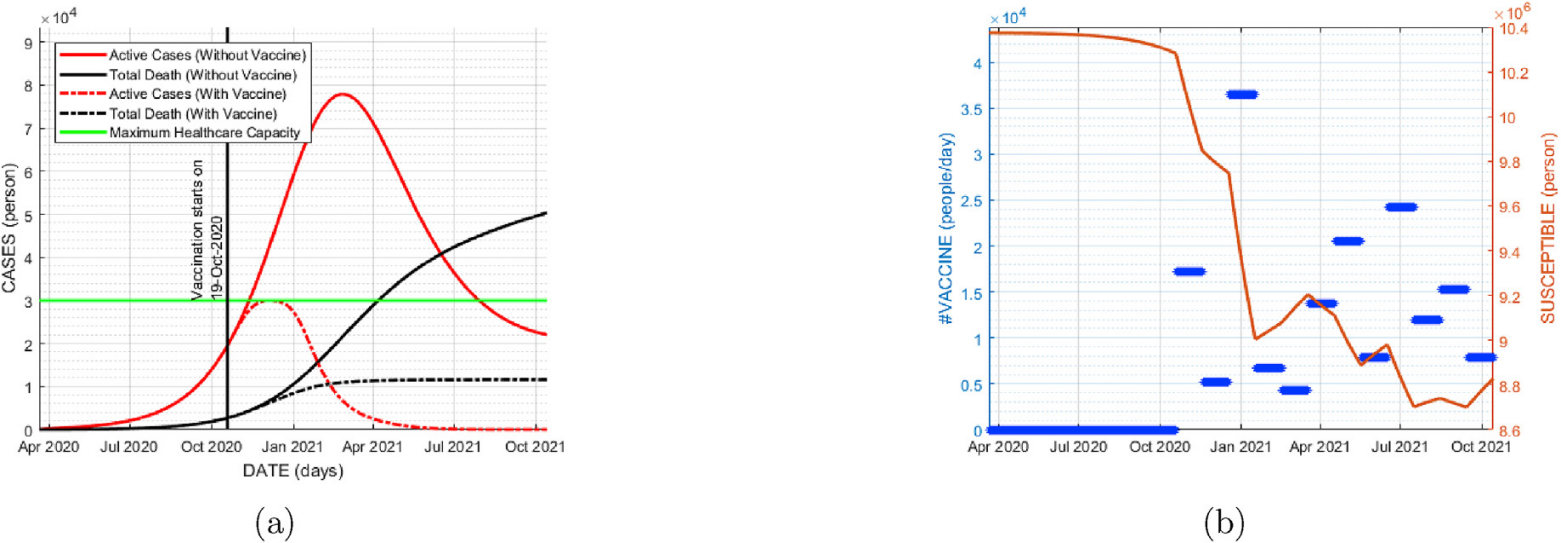
(a) Dynamics of *Q*(*t*) and *D*(*t*) of starting time October 2020 in Jakarta; (b) Number of vaccine per day together with the dynamic of *S*(*t*).

There are many possible *v*(*t*) as the solutions of the optimization problem. Fig. 8(a) and (b) are an example of *v*(*t*) that has a minimum value of Eq. (6) for Jakarta. Fig. 8(a) shows the graph of *Q*(*t*) that is successfully maintained under the constraint *K*_1_ = 30, 000.

Fig. 8(b) shows the number of vaccination per day in Jakarta, represented by the blue thick lines. The number of susceptible people plotted in orange was drastically decreasing only after three periods of vaccination. In Table 5, we can see that none of the constraints is violated. The total number of vaccinated people is about 5.14 million, which is only 49.6% of Jakarta's population. This scenario looks promising where the remaining active cases is only a single person at the final time *t*_*V*_, or a year later. Unfortunately, this scenario is impossible to apply since in fact the vaccine is not ready yet. Some news media predicted the readiness of the vaccine is not earlier than January 2021.

**Table 5:** Estimated results of vaccination scheme starting in Oct 2020 in Jakarta.

Period	Vaccination rate	#Active cases	#Vaccinated	%vaccinated
1	0.0017	28824	516500	4.9%
2	0.0005	29546	672300	6.5%
3	0.0039	21151	1768500	17.1%
4	0.0007	9029	1968200	18.9%
5	0.0005	3689	2098300	20.2%
6	0.0015	1603	2509200	24.2%
7	0.0023	258	3125500	30.1%
8	0.0009	630	3362400	32.4%
9	0.0027	214	4089500	39.4%
10	0.0014	69	4447900	42.9%
11	0.0018	19	4906000	47.2%
12	0.0009	1	5143100	49.6%

**Fig. 9:**
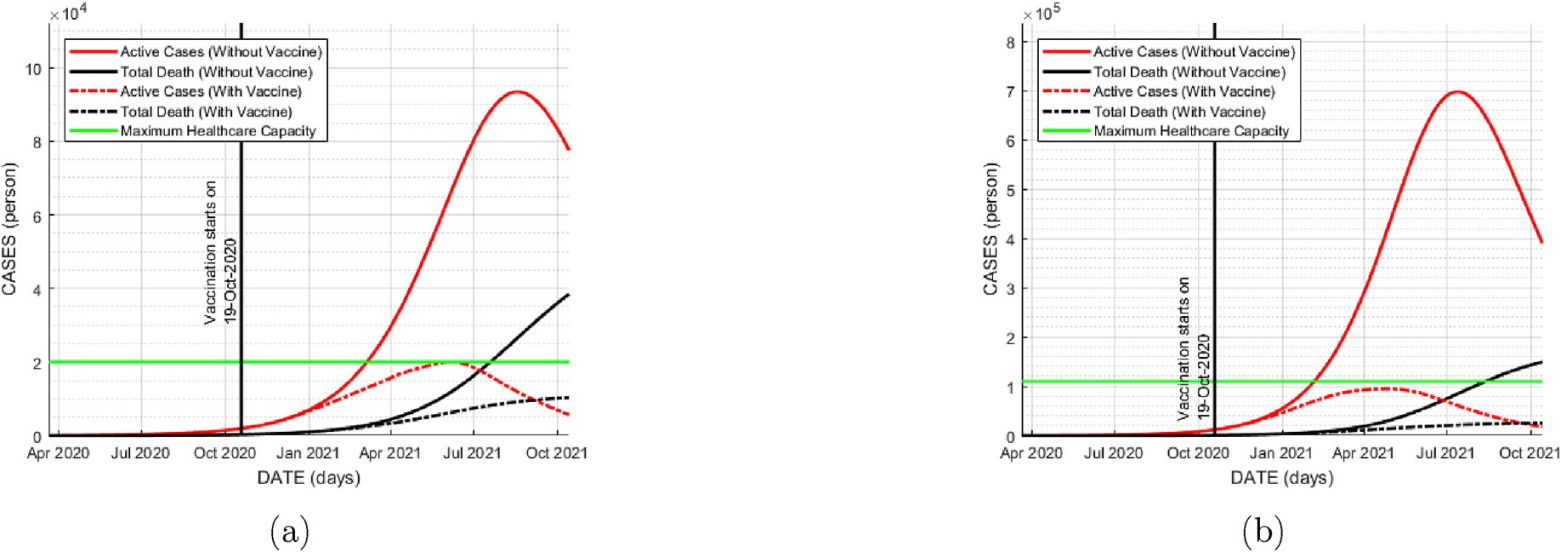
(a) Dynamics of *Q*(*t*) and *D*(*t*) with vaccines given in October 2020 in Banten assuming that *K*_2_ = 34.000; (b) Dynamics of *Q*(*t*) and *D*(*t*) with vaccines in October 2020 in West Java assuming that *K*_2_ = 100.000.

Fig. 9(a) and (b) show the number of active cases for Banten and West Java. It shows that the number of people needed to be vaccinated is about 2.9 million people or 23.3% of the population in Banten, and 12.5 million people or 26% of the population in West Java. Tables representing the estimation results of vaccination scheme for Banten and West Java are given in the Appendices.

If the starting time is in January 2021, Figs. 10(a)-(b) and Table 6 give the results for Jakarta. It shows that the number of susceptible people rapidly decreases only after the second period of vaccination. The fluctuating number of vaccination per day scheduled for each period, plotted as the blue thick line, is one of the solutions found from the approximation process. It is interesting to see whether a constant rate of vaccination will be effective or not, because it will be simpler to operate in the real condition. We have this kind of scenario later in this paper.

**Fig. 10:**
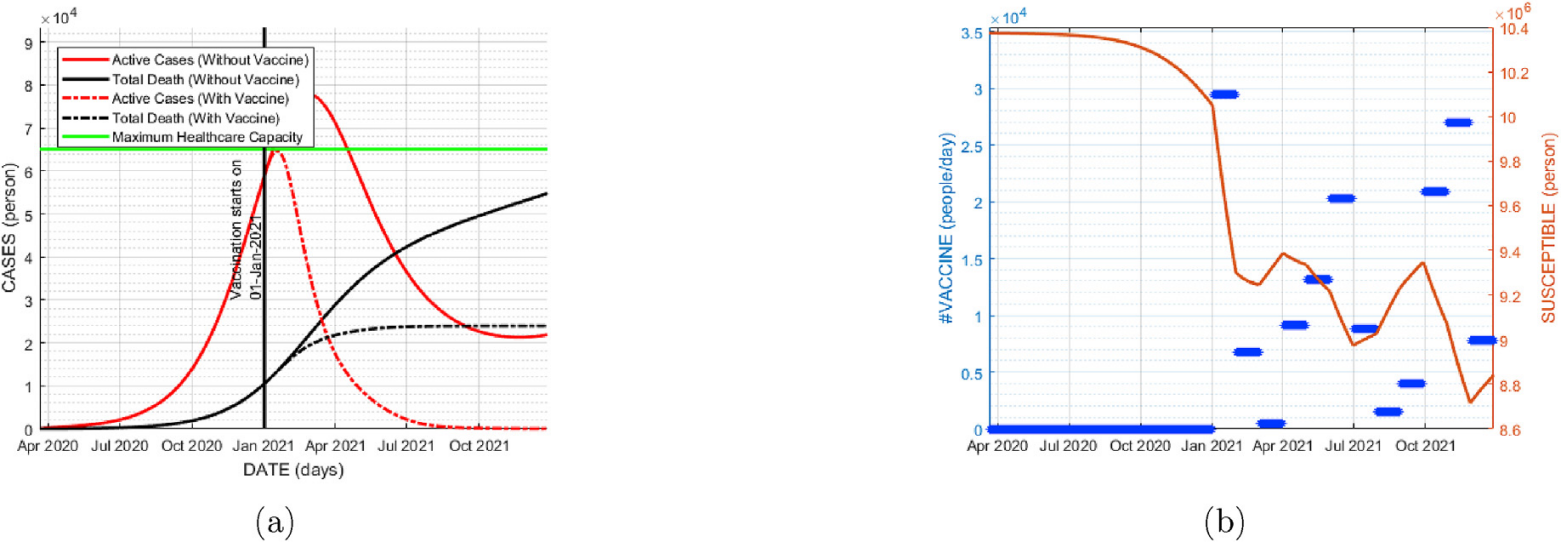
(a) Dynamics of *Q*(*t*) and *D*(*t*) with vaccine on January 2020 in Jakarta; (b) Number of vaccine per day together with the dynamic of *S*(*t*).

**Table 6:** Estimation results of vaccination scheme starting in Jan 2021 in Jakarta.

Period	Vaccination rate	#Active cases	#Vaccinated	%Vaccinated
1	0.0031	59515	884500	8.5%
2	0.0006	34029	1088200	10.5%
3	0.0001	17463	1103400	10.6%
4	0.0010	9578	1378500	13.2%
5	0.0014	5009	1773500	17.1%
6	0.0022	2242	2380700	22.9%
7	0.0010	840	2646400	25.5%
8	0.0002	326	2691200	25.9%
9	0.0004	152	2809400	27.1%
10	0.0023	74	3437400	33.2%
11	0.0030	28	4245100	40.9%
12	0.0009	1	4475400	43.2%

**Fig. 11:**
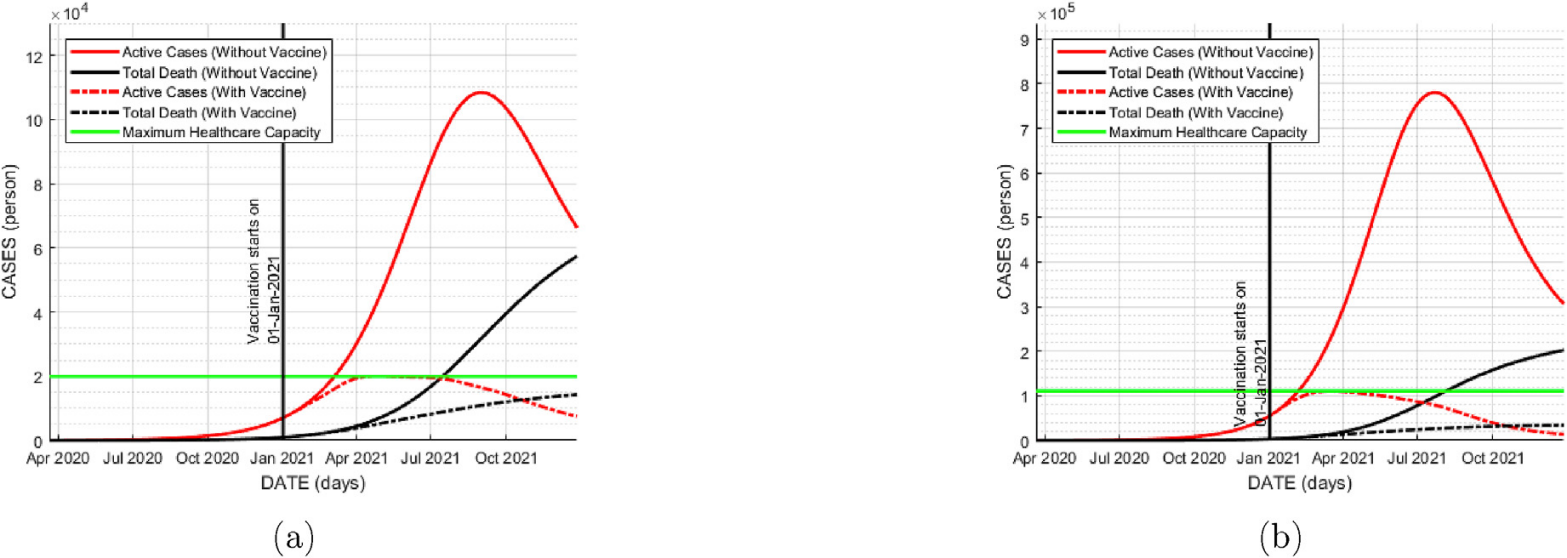
Dynamics of *Q*(*t*) and *D*(*t*) with vaccine in January 2021 in (a) Banten; (b) West Java.

From Table 6, the number of vaccinated people is 4.48 million or 43.2% of the population in Jakarta. The vaccination is successful because the remaining active case is only one. The simulations of the vaccination for Banten and West Java are given in Fig. 11, where the number of active cases is limited to the value of *K*_1_. The number of vaccinated people is 2.1 million or 17% of the population in Banten, and 10.1 million or 22.7% of the population in West Java.

**Fig. 12:**
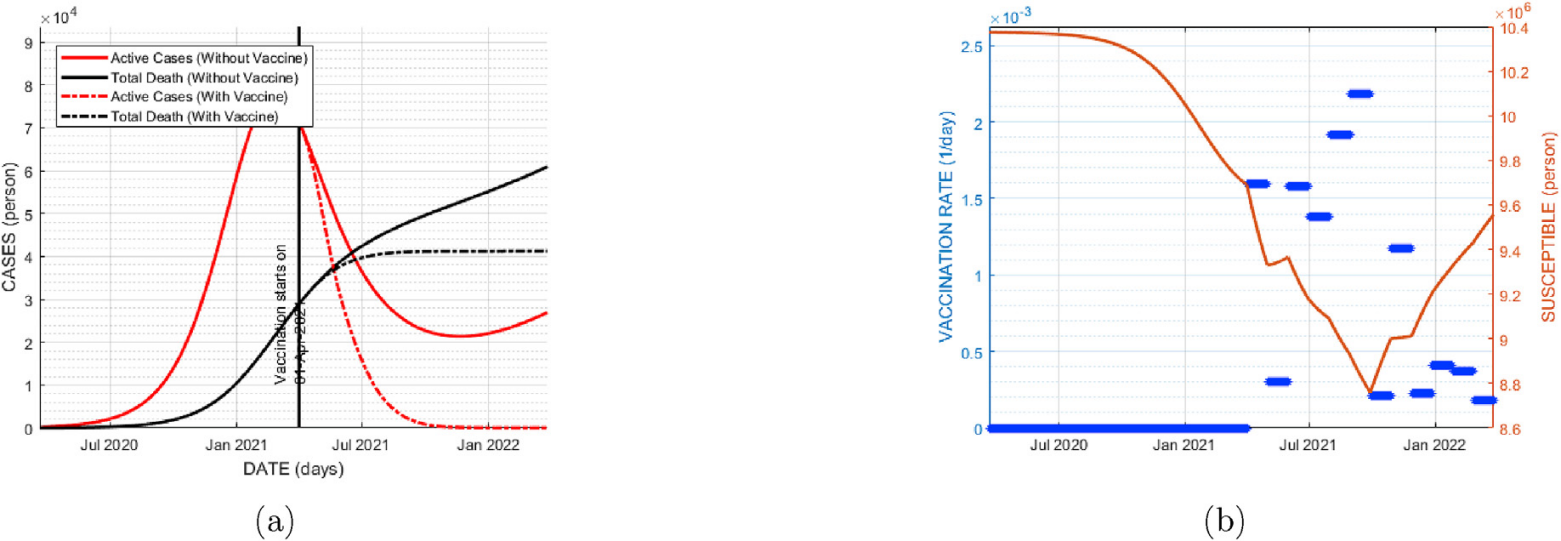
(a) Dynamics of *Q*(*t*) and *D*(*t*) with vaccine on April 2021 in Jakarta; (b) Number of vaccine per day together with the dynamic of *S*(*t*).

Now the starting time of vaccination is after the number of active cases reaches its peak. The starting time is therefore different for each province, i.e., April 2021 in Jakarta, October 2021 in Banten, and September 2021 in West Java. Fig. 12(a) shows a steeper decrease of *Q*(*t*) than that of without vaccination in Jakarta. The number of death *D*(*t*) seems to stabilize to about 4,000 as the peak of outbreak happened before the vaccination. From Table 7, the total number of people to be vaccinated is 3.19 million or 30.4% of population in Jakarta. If we compare between Fig. 8, Fig. 10, Fig. 12, the total numbers of death are increasing, which means that the later the starting time of vaccination, the higher the number of the pandemic casualties.

**Table 7:** Estimation results of vaccination scheme starting in April 2021 in Jakarta.

Period	Vaccination rate	#Active cases	#Vaccinated	%Vaccinated
1	0.0016	53327	453600	4.3%
2	0.0003	30624	537400	5.2%
3	0.0016	16166	976900	9.4%
4	0.0014	7285	1355800	13.1%
5	0.0019	2863	1874500	18.1%
6	0.0022	946	2454600	23.7%
7	0.0002	284	2510400	24.2%
8	0.0012	96	2827000	27.2%
9	0.0002	36	2888000	27.8%
10	0.0004	16	3001000	28.9%
11	0.0004	9	3106000	29.9%
12	0.0002	5	3186900	30.4%

Overall, the presence of vaccine jabs can cause a significant drop of susceptible people and hence lift the number of active cases down. The calculated rates of inoculation are varied each month which is based on the definition of *v*(*t*) in Section 2.1 which is too complicated if it comes to the practical implementation. The extremely fluctuating rates of jab can cause extreme addition and subtraction of medical personnel each month. In a response to that, the constant rate of vaccination is further discussed in Section 5.1.3.

#### 5.1.2 Frequency of the vaccination

In Fig. 10(a), vaccination for 12 months can make the number of active cases decrease significantly in the first month of the vaccination period. It is interesting to see whether a one time vaccination in the first month could really work. Most of the time when the first attempt shows a good result, the vaccination program could be immediately stopped to reduce cost.

**Fig. 13:**
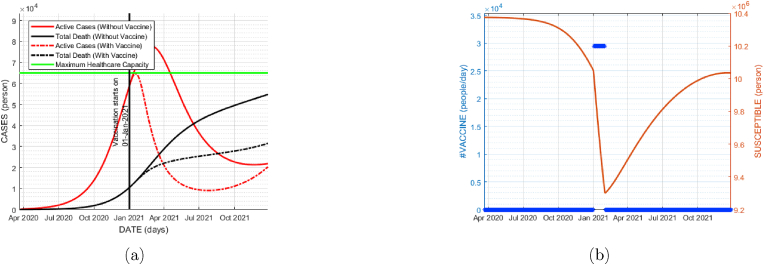
(a) Dynamics of *Q*(*t*) and *D*(*t*) with vaccine only in January 2021; (b) Number of vaccine per day and the dynamic of *S*(*t*).

**Fig. 14:**
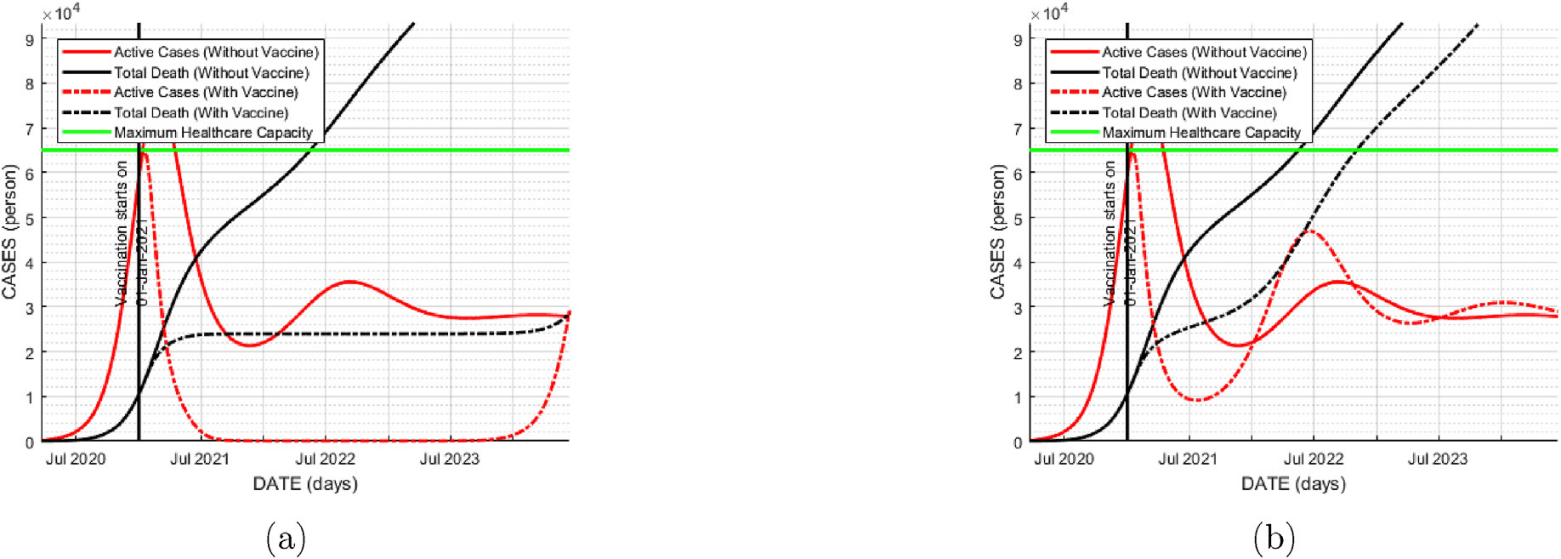
(a) 12-month vaccination; (b) One-month vaccination in Jakarta.

Fig. 13 depicts a simulation of this one-time vaccination for Jakarta, where *v*(*t*) = 0.0031 for time *t* in January 2020, and *v*(*t*) = 0 for the other months. It shows a significant decrease of the active cases at the beginning of vaccination, but it will start to increase from August 2021 onward, which may cause another outbreak in the future. This prediction is clearly seen when we plot the graphs in a longer time-span in Fig. 14. On the left, a vaccination consistently given for 12 months from January 2021 can make active cases disappear until the beginning of year 2024. On the right, a one-time vaccination potentially creates another outbreak in July 2022. The increase of death toll seems to be slowed down about one year, then it will increase to the same number as that obtained from the non-vaccine model with a few month delay.

#### 5.1.3 Constant vaccination rates

The scenarios from the previous subsection assess the timing and frequency of vaccination. The scheme of vaccination obtained from the approximation process gives fluctuating values of vaccination rates that make it hard to be implemented. It means that there will be fluctuating needs of the vaccination workers per period. Now we simulate a the vaccination program with constant rates and analyze the dynamics of the active cases.

**Fig. 15:**
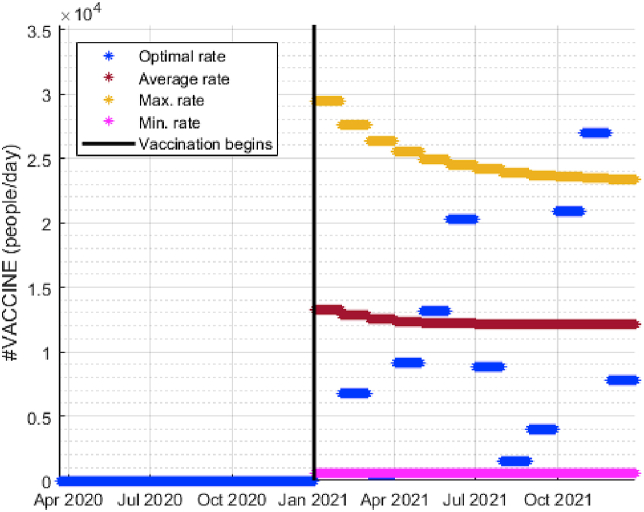
The number of vaccinated people on each period for several schemes: optimum, maximum, average, and minimum of vaccination rate.

There are 3 scenarios with different values of constant rates starting from January 2021 based on the optimal vaccination rates obtained in Table 6. The constant vaccination rate for the first, second and third scenarios are the average of rates ($\bar{c}=0.0014$), the maximum rate (*c*_*max*_ = 0.0031), and the minimum rate (*c*_*min*_ = 0.0005), respectively. The number of vaccinated people of those scenarios are plotted in Fig. 15, where the original optimum values, the maximum, the average and the minimum rates are respectively represented by blue stripes, upper magenta stripes, middle magenta stripes, and lower magenta lines. Due to the decreasing number of susceptible people, the number of vaccinated ones is also decreasing, even though the vaccination rates are constant.

**Fig. 16:**
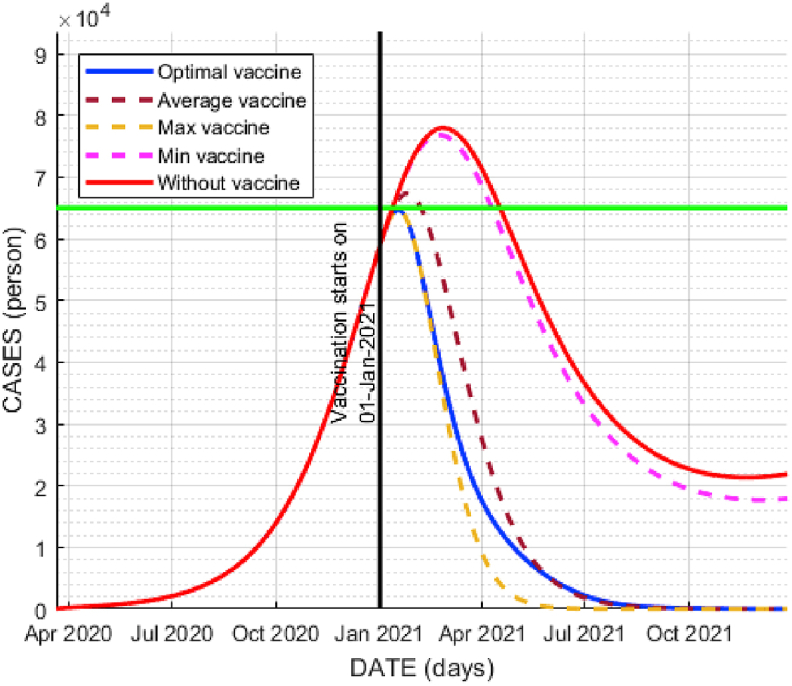
The dynamics of *Q*(*t*) using the constant value of vaccination rate.

Let the optimum fluctuated rates be the benchmark of the uses of the healthcare facility that requires a cost calculated from Eq. (6). The expenses calculated from implementing those scenarios will be compared to this benchmark expense. Fig. 16 shows the active cases *Q*(*t*) from all scenarios where the optimum vaccination rate is plotted in solid blue line. The average rate scenario gives values *Q*(*t*) exceeding the maximum healthcare capacity (the green line) and it requires 99.1% of the benchmark cost. The maximum rate scenario gives a plot of *Q*(*t*) that resembles the plot from the optimum rates, but it requires almost twice of the benchmark cost, i.e. 192%. Finally, the minimum rate scenario gives insignificant reduction of *Q*(*t*) compared to the plot of *Q*(*t*) without vaccination. It concludes that the optimum fluctuated rates are the best scheme among other constant rate scenarios based on the dynamic of active cases and the required cost.

#### 5.1.4 Discussion

Considering the simulations for different starting time scenarios, the vaccination program ideally should start in the middle of the pandemic, which is October 2020. The total number of deaths can be suppressed significantly. Unfortunately, this scenario is impossible to apply since the vaccine was not ready yet at that time. If the beginning of the vaccination program is delayed, the required amount of vaccines will decrease. It therefore seems better to begin a vaccination program after the peak of the outbreak. However, delaying the starting time will result in increasing total deaths. In fact, the decrease of the total number of deaths is not significant if we choose the latest scenario.

Having simulated different frequency of vaccination, it shows in Fig. 13(a) that only a single time of vaccination gives insignificant reduction of the number of active cases at the end of the intended vaccination program. Moreover, the final number of infectious people in the simulation, *I*(*t*_*V*_), is about 800 people in Jakarta, which is still too high that could trigger another peak of outbreak through reinfection. It is concluded that a consistent schedule of vaccination is necessary to significantly reduce the number of active cases.

The pattern of the vaccination rate is also interesting to observe. Having seen the results from the first two scenarios, where the vaccine was applied before the number of active cases reaches its peak, the vaccination rate obtained from the optimization procedure is high at the beginning of the vaccination schedule. It seems that the healthcare facility is not yet at maximum capacity *K*_1_, so much effort can be used to reduce the number of active cases by maximizing the vaccination shots per day. On the other hand, if the vaccine is applied after the peak of outbreak, the vaccination rate will be low at the beginning and then high at the end of the vaccination. In this scenario, the healthcare facility is already at the maximum capacity, so the main focus is preventing another peak of outbreak to come.

### 5.2 Age-structured model

Now four scenarios of prioritizing particular age groups are implemented on the age-structured model, where the priority is respectively given to groups of the active and older people, groups of 20 years old and younger, group of active people only, or alternating targeted group in each period. The starting time is January 2020 and we use the scheme of vaccination rates shown in Fig. 10.

#### 5.2.1 Groups of active and older adults (above 50)

**Fig. 17:**
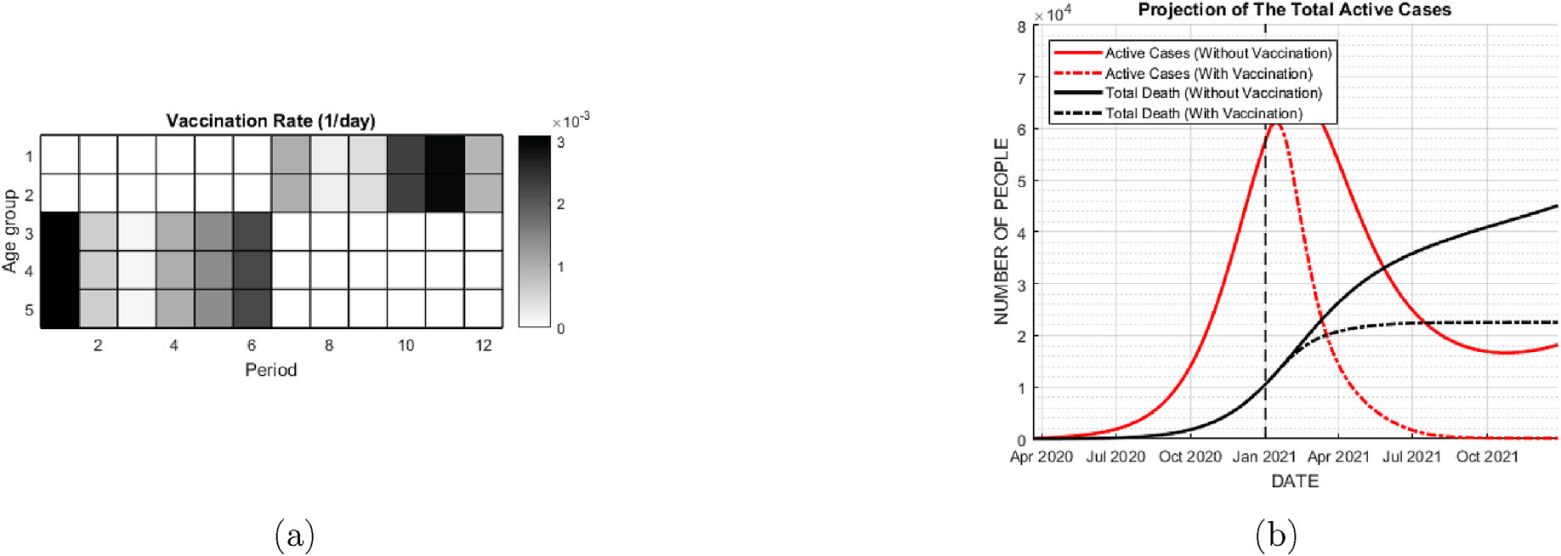
Vaccination scenario by prioritizing active and older adults (above 50) (a) Vaccination schedule; (b) Dynamics of *Q*(*t*) and *D*(*t*) in Jakarta.

**Fig. 18:**
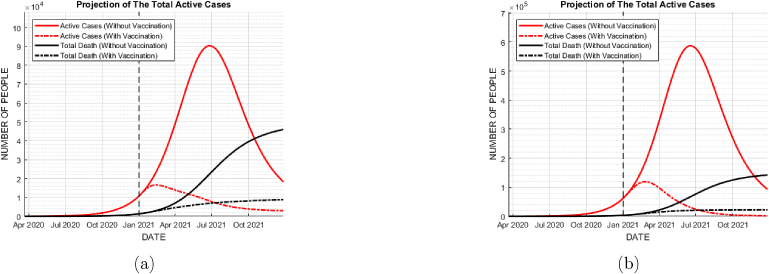
Vaccination scenario by prioritizing active and older adults (above 50) in (a) Banten; (b) West Java.

During the first six months, vaccine shots are given to workers and high-risk people, which are 20 and older, then in the remaining months, they are given to younger groups. As seen in Fig. 17(a), the darker color of the cells for certain periods means higher rate of vaccination. Fig. 17(b) illustrates the simulation result of the total numbers *Q*(*t*) and *D*(*t*) of all age-groups using this scenario in Jakarta. The simulation results from Banten and West Java are given in Fig. 18. The vaccination performs well in reducing the numbers of active cases and death toll.

#### 5.2.2 Groups of 20 years old and younger persons

**Fig. 19:**
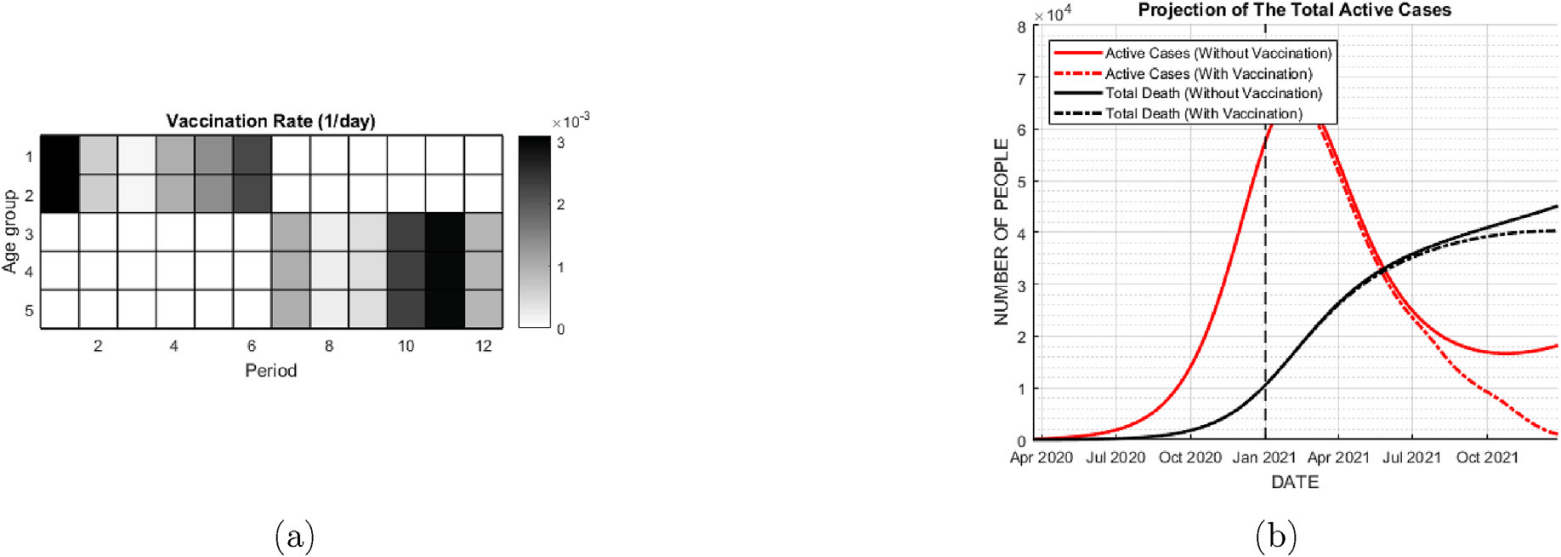
Vaccination scenario by prioritizing 20 years old and younger (a) Vaccination schedule; (b) Dynamics of *Q*(*t*) and *D*(*t*) in Jakarta.

**Fig. 20:**
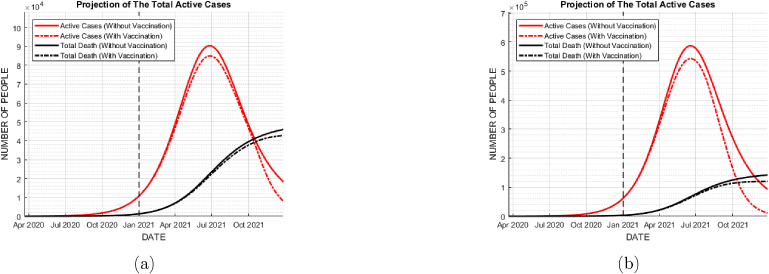
Vaccination scenario by prioritizing 20 years old and younger in (a) Banten; (b) West Java.

In this scenario, the first interval of six months is the vaccination time only for the younger people of age 0–19, and the remaining time is for the active and older people's groups, as scheduled in Fig. 19(a). The dynamics of the total numbers *Q*(*t*) and *D*(*t*) of all age-groups using this scenario in Jakarta, Banten and West Java are described in Fig. 19, Fig. 20, respectively. It seems that this scenario gives insignificant decrease on the total numbers of active cases and death toll.

#### 5.2.3 Alternating targeted group

**Fig. 21:**
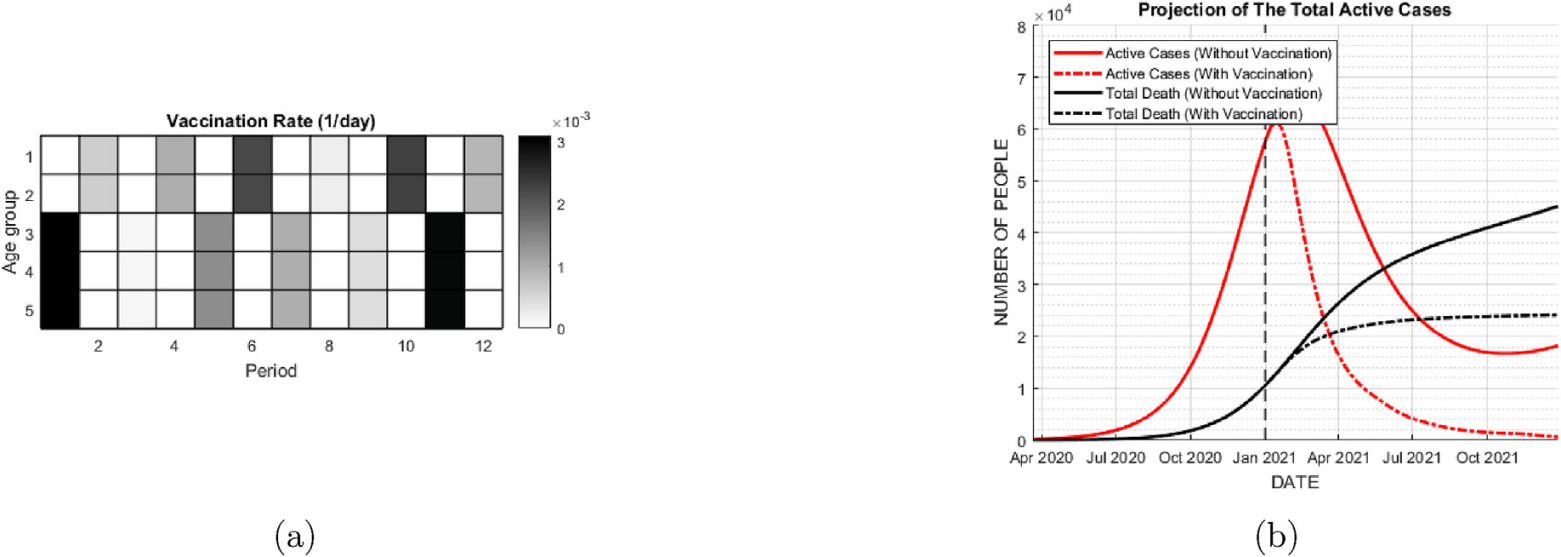
Alternating vaccination by switching vaccination target in each period (a) Vaccination schedule; (b) Dynamics of *Q*(*t*) and *D*(*t*) in Jakarta.

**Fig. 22:**
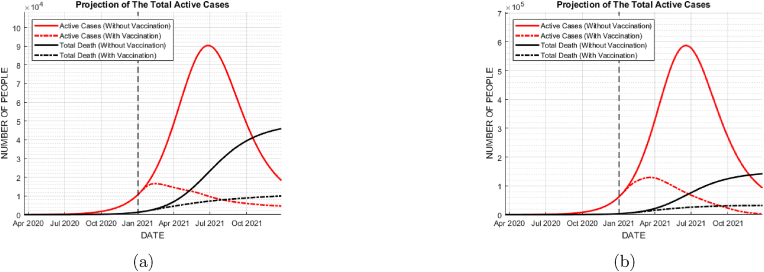
Alternating vaccination target in each period in (a) Banten; (b) West Java.

In this scenario, we change the target of age-groups in certain ways that is shown in Fig. 21(a). High rate vaccination in the first month is given to the active and older people, i.e., age 20 and older. Fig. 21, Fig. 22 describe the dynamics of the total number *Q*(*t*) and *D*(*t*) of all age-groups obtained using this scenario in Jakarta, Banten, and West Java. Compared to those obtained by prioritizing active and older people, this scenario yields a thicker tail in the active cases. This observation can be seen in every region we observed. Thus, this scenario is not likely preferable than the first one, i.e., prioritizing the active and older people.

#### 5.2.4 Only the active people

**Fig. 23:**
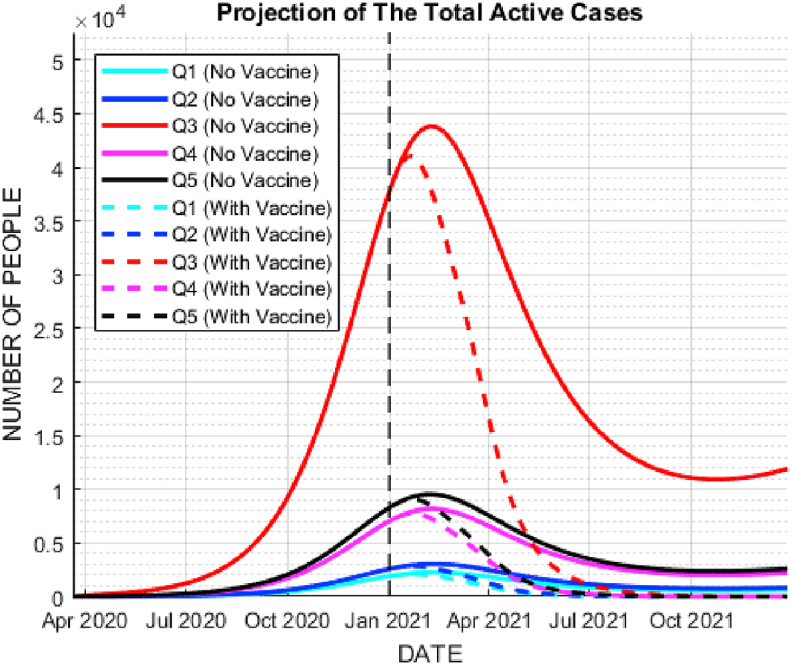
Dynamics of *Q*_*i*_(*t*) once the third group of age in Jakarta is vaccinated.

**Fig. 24:**
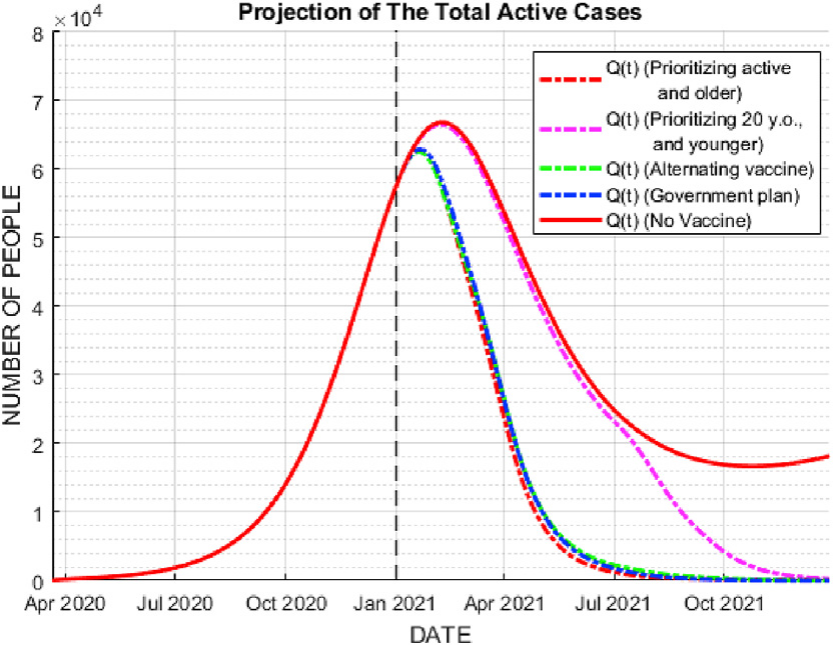
Comparison of *Q*(*t*) of all the observed vaccination scenarios in Jakarta.

**Fig. 25:**
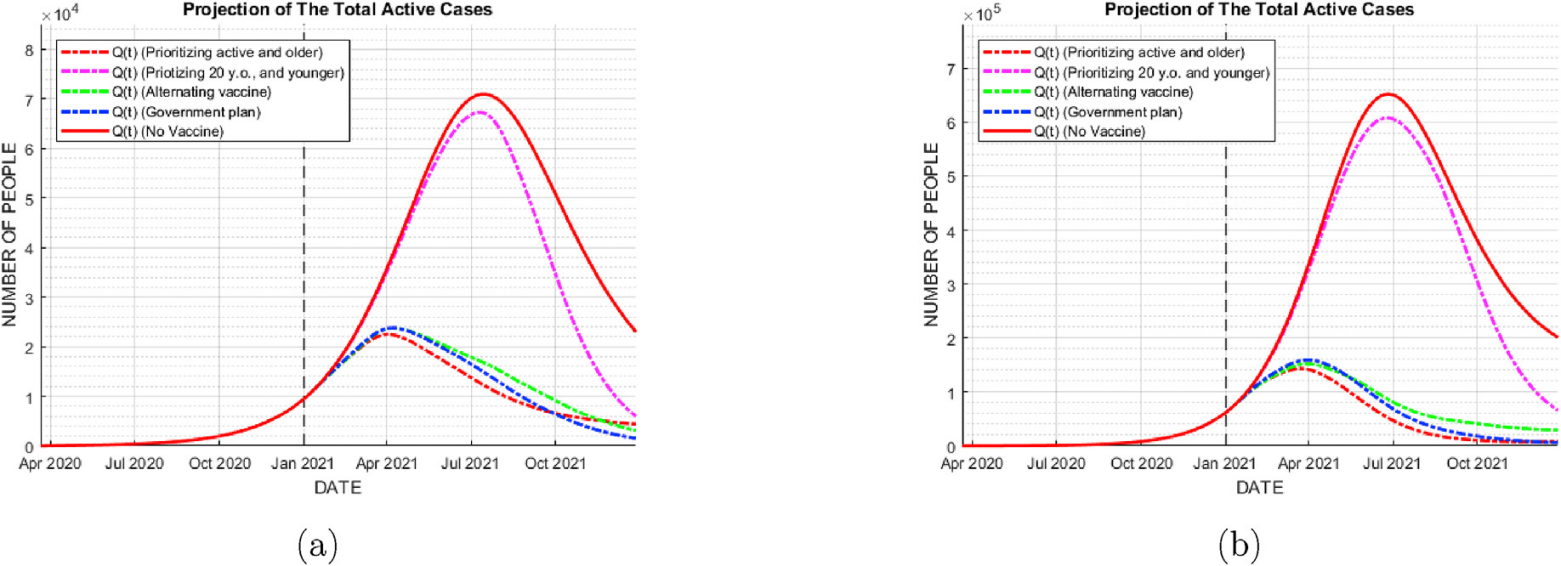
Comparison of *Q*(*t*) of all the observed vaccination scenarios in (a) Banten; (b) West Java.

Assuming that the Indonesian government plans to conduct vaccination only to the group of active people, i.e., age 20–49, we perform simulations in this scenario. Fig. 23 depicts the dynamics of *Q*_*i*_(*t*) once the third age-group is vaccinated. On the other hand, we also provide the dynamics of active cases that results from the other three scenarios as a comparison. Fig. 24 shows that vaccination prioritizing the active and older people is more preferable due to its significant reduction of cases. This argument is also valid to the results given in Banten and West Java as depicted by Fig. 25.

#### 5.2.5 Discussion

Our simulations show that the vaccination scenario by first targeting the active and older adults (above 50) (group 3–5) is better than the other considered scenarios. The numbers of active cases and total deaths decreased significantly. In the result of scenario 2, the decrease is insignificant, because the transmission rate of virus among the 20-year-old and younger people is much lower than that of the other age groups, as shown in Table 2.

After conducting simulations by targeting the active people only, we obtain that the dynamics of the number of active cases from the other age groups, *Q*_*i*_(*t*), *i* ≠ 3, also decreased once people in the third age-group has been vaccinated. It is understandable because the key of the disease transmission is the contact among people. As the active people have larger access to people from the other age-groups, the transmission across age groups becomes highly possible. Once the biggest source of infectious people has been vaccinated, the number of active cases in the other groups of age can be expected to decrease significantly.

## 6 Conclusions and recommendations

Having constructed the *SIQRD* model with and without age structures and developed several scenarios on the implementation of a vaccination program, we concluded our work in the following findings that are related to the proposed questions:

• Modifying the *SIRD* into the *SIQRD* model by adding quarantine, reinfection, and even vaccination aspects has been considered to be capable in representing how COVID-19 spreads in several provinces in Indonesia. Utilizing the existing data and information related to those provinces, the graphs of the simulations well resemble the corresponding figure in the real situation.
• Vaccination should be implemented in the early stage of the pandemic. This is to suppress the number of active cases immediately, and consequently the total deaths. After the active cases reach their peaks, vaccination does not reduce the total deaths significantly.
• Vaccination should be implemented consistently following a schedule for a certain period. An implementation for only one or two months does not reduce the number of infectious persons, and eventually it will fail to prevent another outbreak.
• Prioritizing the active and older adults (above 50) for vaccination over others and prioritizing the active people only reduce significantly the total deaths.

## Declaration of competing interest

The authors declare that they have no known competing financial interests or personal relationships that could have appeared to influence the work reported in this paper.

## Figures and Tables

**Fig. A.26:**
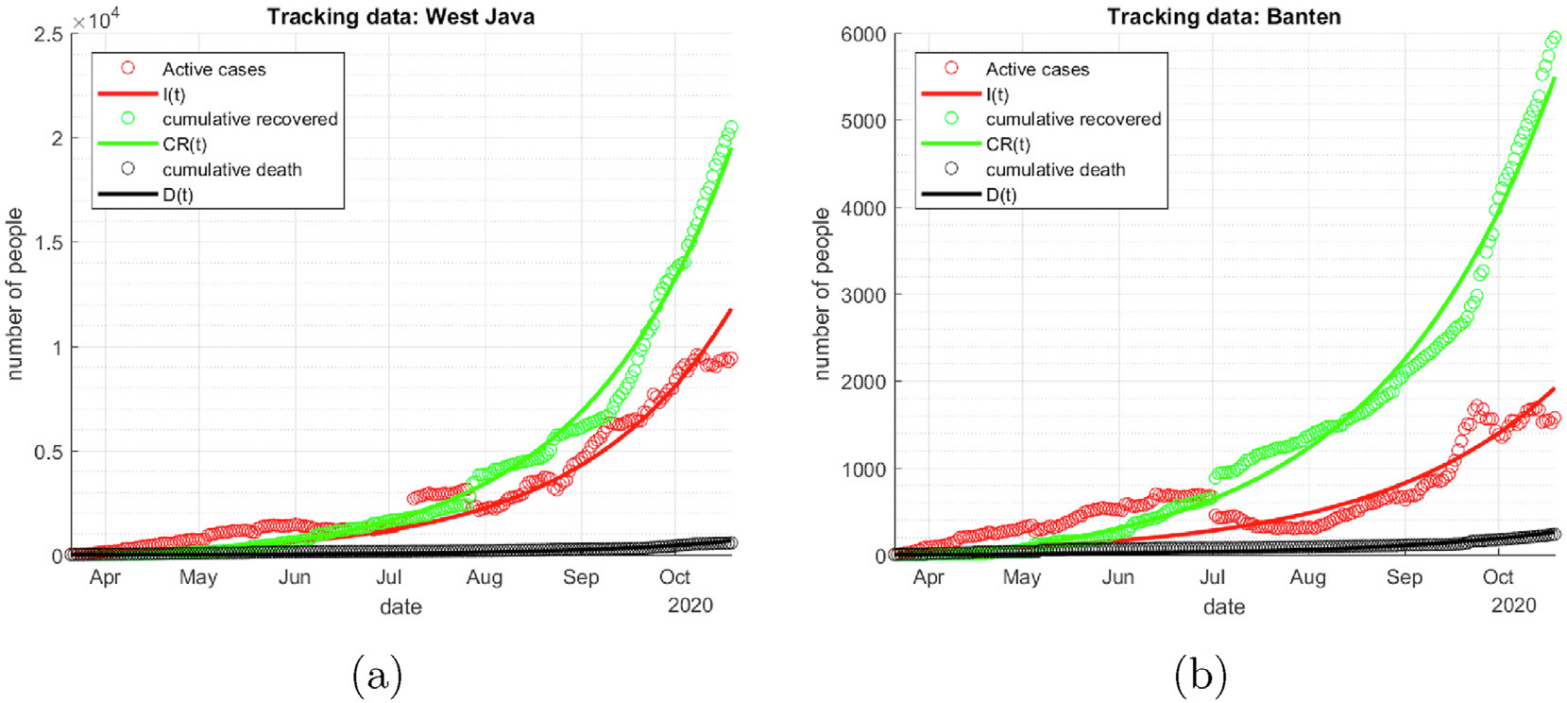
Fitting results of the non-age-structured model in: (a) Banten; (b) West Java

**Table A.8:** Parameter estimation of the non-age-structured model for Jakarta

Region	*β*	*γ*	*δ*	*I*(*t*_0_)	*R*_0_
Banten	0.5968	0.0520	0.0024	9	1.0447
West Java	0.6016	0.0354	0.0012	20	1.0528

**Table B.9:** Obtained values *β*_*i*_ for the age-structured model of Banten and West Java

Region	*β*_1_	*β*_2_	*β*_3_	*β*_4_	*β*_5_
Banten	0.0253	0.0358	0.1131	0.1703	0.4525
West Java	0.0262	0.0371	0.1172	0.1765	0.4690

**Table C.10:** Estimation results of vaccination scheme starting in Oct 2020 in Banten

Period	Vaccination rate	#Active cases	#Vaccinated	%vaccinated
1	0.0001	3264	8300	0.6%
2	0.0004	5333	171900	1.4%
3	0.0007	7742	422300	3.4%
4	0.0004	9859	554200	4.5%
5	0.0002	11777	623100	5.0%
6	0.0006	13516	834300	6.7%
7	0.0001	14626	868300	6.9%
8	0.0019	14510	1541700	12.4%
9	0.0008	11271	1824300	14.6%
10	0.0003	7886	1933200	15.3%
11	0.0007	5510	2183700	17.5%
12	0.0021	3590	2898400	23.3%

**Table C.11:** Estimation results of vaccination scheme starting in Jan 2021 in Banten

Period	Vaccination rate	#Active cases	#Vaccinated	%vaccinated
1	0.0007	11158	242500	2.1%
2	0.0002	15906	318500	2.5%
3	0.0012	19364	767000	6.2%
4	0.0002	19984	842800	6.7%
5	0.0001	19731	892100	7.2%
6	0.0014	19468	1042300	8.4%
7	0.0006	18434	1267200	10.1%
8	0.0002	16524	1354600	10.8%
9	0.0008	14436	1635000	13.1%
10	0.0004	11873	1782800	14.3%
11	0.0006	9512	1996200	16.1%
12	0.0004	7474	2130200	17.1%

**Table C.12:** Estimation results of vaccination scheme starting after the peak of outbreak in Banten

Period	Vaccination rate	#Active cases	#Vaccinated	%vaccinated
1	0.0001	70842	3000	0.1%
2	0.0002	58624	68800	0.5%
3	0.0003	47501	176900	1.4%
4	0.0002	37728	231600	1.9%
5	0.0001	30432	259600	2.1%
6	0.0002	25370	353500	2.8%
7	0.0003	21704	367500	2.9%
8	0.0008	18818	655600	5.3%
9	0.0004	15324	790200	6.3%
10	0.0001	12330	838000	6.7%
11	0.0003	10262	955200	7.7%
12	0.0008	8503	1258900	10.1%

**Table C.13:** Estimation results of vaccination scheme starting in Oct 2020 in West Java

Period	Vaccination rate	#Active cases	#Vaccinated	%vaccinated
1	0.0001	22221	27000	0.1%
2	0.0019	37253	2651000	5.5%
3	0.0017	44647	5003000	10.4%
4	0.0001	41397	5200000	10.8%
5	0.0001	41393	5330000	11.1%
6	0.0018	36772	7795000	16.2%
7	0.0009	24604	8978000	18.6%
8	0.0010	16970	10290000	21.4%
9	0.0008	10977	11277000	23.5%
10	0.0007	6910	12193000	25.4%
11	0.0001	4404	12306000	25.7%
12	0.0001	3046	12430000	25.9%

**Table C.14:** Estimation results of vaccination scheme starting in Jan 2021 in West Java

Period	Vaccination rate	#Active cases	#Vaccinated	%vaccinated
1	0.0010	92140	2187000	4.5%
2	0.0008	117610	3333000	6.9%
3	0.0012	122050	4921000	10.2%
4	0.0006	107180	5678000	11.8%
5	0.0001	88060	5879000	12.2%
6	0.0011	71690	7354000	15.3%
7	0.0010	54550	8623000	17.9%
8	0.0005	38940	9295000	19.3%
9	0.0002	27710	9533000	19.8%
10	0.0008	20560	10584000	22.0%
11	0.0002	15610	10804000	22.5%
12	0.0001	12710	10829000	22.7%

**Table C.15:** Estimation results of vaccination scheme starting after the peak of outbreak in West Java

Period	Vaccination rate	#Active cases	#Vaccinated	%vaccinated
1	0.0001	676430	22900	0.1%
2	0.0004	548920	495200	1.1%
3	0.0006	409940	1235900	2.5%
4	0.0003	286400	1621700	3.3%
5	0.0002	197270	1822400	3.8%
6	0.0005	139270	2461500	5.1%
7	0.0001	101840	2561500	5.3%
8	0.0014	76470	4480700	9.3%
9	0.0007	53650	5363600	11.1%
10	0.0003	25220	5697800	11.8%
11	0.0006	17400	6482300	13.4%
12	0.0016	5023	8558300	17.8%

## Appendix A Graphs of non age-structured model

For Banten and West Java, the comparisons of simulated and real data are following.

Those figures above are depicted by the estimation results of *β*, *γ*, *δ*, and *I*(*t*_0_) for Banten and West Java. The obtained parameters of the non age-structured model are given by the following table.

## Appendix B Estimated values of transmission rate

We provide tables representing Obtained values *β*_*i*_ for the age-structured model of Banten and West Java.

## Appendix C Vaccination scheme

We provide tables representing the estimation results of the vaccination scheme for Banten and West Java with several different starting times.

1. Banten
  • Vaccination in October 2020;

• Vaccination in January 2021;

• After the peak of outbreak.

2. West Java
  • Vaccination in October 2020;

• Vaccination in January 2021;

• After the peak of outbreak.
